# The Andean Latin-American burden of diabetes attributable to high body mass index: A comparative risk assessment

**DOI:** 10.1016/j.diabres.2019.107978

**Published:** 2020-02

**Authors:** Rodrigo M. Carrillo-Larco, Jonathan Pearson-Stuttard, Antonio Bernabe-Ortiz, Edward W. Gregg

**Affiliations:** a Department of Epidemiology and Biostatistics, School of Public Health, Imperial College London, London, UK; b CRONICAS Centre of Excellence in Chronic Diseases, Universidad Peruana Cayetano Heredia, Lima, Peru; c MRC-PHE Centre for Environment and Health, School of Public Health, Imperial College London, London, UK; d Universidad Científica del Sur, Lima, Peru

## Abstract

**Background:**

Body mass index (BMI) has increased in Latin-America, but the implications for the diabetes burden have not been quantified. We estimated the proportion and absolute number of diabetes cases attributable to high BMI in Bolivia, Ecuador and Peru (Andean Latin-America), with estimation of region-level indicators in Peru.

**Methods:**

We estimated the population attributable fraction (PAF) of BMI on diabetes (regardless of type 1 or 2) from 1980 to 2014, including the number of cases attributable to overweight (BMI 25–<30), class I (30–<35), class II (BMI 35–<40) and class III (BMI ≥ 40) obesity. We used age- and sex-specific prevalence estimates of diabetes and BMI categories (NCD-RisC and Peru’s DHS survey) combined with relative risks from population-based cohorts in Peru.

**Findings:**

Across Andean Latin-America in 2014, there were 1,258,313 diabetes cases attributable to high BMI: 209,855 in Bolivia, 367,440 in Ecuador and 681,018 in Peru. Between 1980 and 2010, the absolute proportion of diabetes cases attributable to class I obesity increased the most (from 12.9% to 27.2%) across the region. The second greatest increase was for class II obesity (from 3.6% to 16.5%). There was heterogeneity in the fraction of diabetes cases attributable to high BMI by region in Peru, as coastal regions had the largest fractions, and so did high-income regions.

**Interpretation:**

Over one million diabetes cases are attributable to high BMI in Andean Latin-America. Public health efforts should focus on implementing population-based interventions to reduce high BMI and to develop focused interventions targeted at those at highest risk of diabetes.

## 1 Introduction

The global burden of high body mass index (BMI) is well established and quantified in low and middle-income countries [1]. High BMI is the leading cause of type 2 diabetes. This is particularly true for Latin-America where diabetes prevalence has increased [2], the diabetes-associated mortality risk is higher than in any other world region [3], and diabetes is a significant threat to health systems, economy and population health [4], [5]. The exact mechanisms underpinning the high BMI and diabetes association may vary by geographic region and different populations may have increased risk of diabetes at different BMI levels. Furthermore, BMI is associated with diabetes in a non-linear fashion, as people with severe obesity account for a disproportionate burden. However, the relative contribution of severe obesity (e.g., class III obesity) versus obesity or overweight to the growth of the diabetes burden has not been quantified for Latin-America.

While global public health agencies (e.g., World Health Organization, WHO) and their regional counterparts (Pan American Health Organization, PAHO) have prioritized prevention and management of high BMI (≥25 kg/m^2^) and diabetes [6], [7], [8], [9], and fostered surveillance systems at the population level to inform policies and evaluations [10], [11], the magnitude, variation and impact of high BMI upon diabetes in Latin-America and Andean Latin-America (Bolivia, Ecuador and Peru) are largely unknown. To inform health policies and monitoring systems in Andean Latin-America, we estimated the population attributable fraction (PAF) and absolute number of diabetes cases attributable to high BMI, including stratification by high BMI and obesity category.

## 2 Methods

### 2.1 Study overview

We integrated global population-based prevalence estimates of diabetes (regardless of type 1 or 2) and BMI (available through NCD-RisC) [2], [12], along with relative risks (RR) estimating the association between high BMI and diabetes derived from population-based cohorts in Peru [13], [14], to estimate the fraction and absolute number of diabetes cases attributable to high BMI in Bolivia, Ecuador and Peru (Andean Latin-America). We also used nationally representative data on BMI and diabetes prevalence from Peru to make estimates for all twenty-five regions in Peru. Details about the data sources and analysis are available in supplementary material pp. 04–09.

### 2.2 Data components

In a systematic search we looked for prospective cohort studies that assessed the association between BMI and diabetes without restriction to type 1 or type 2 diabetes mellitus. Regional expert knowledge was also used to retrieve additional data sources. To the best of our knowledge, three cohort studies have assessed the association between BMI and diabetes in Andean Latin-America [13], [14], [15], and two of these were accessed and re-analysed for this study [13], [14]. These were population-based cohorts conducted with people aged ≥30 years in Peru.

The risk estimates retrieved from these Peruvian cohorts were applied to the other Andean Latin-American countries, Bolivia and Ecuador. These are middle-income countries where death rates of major non-communicable diseases, as well as prevalence estimates of diabetes and obesity are comparable (supplementary material p. 03) [2], [12], [16]. To further support this decision, other global population health metrics endeavours such as the Global Burden of Disease study (GBD) as well as the Non-communicable Diseases Risk Factor Collaboration (NCD-RisC) [1], [2], [12], group Bolivia, Ecuador and Peru under the Andean Latin-America umbrella.

We estimated the PAF of diabetes cases attributable to high BMI in Bolivia, Ecuador and Peru, where the optimal BMI distribution was set at <25 Kg/m^2^. Furthermore, we also estimated the PAF of diabetes cases attributable to high BMI in each of the 25 regions in Peru as an example of the variability of these estimates within an Andean Latin-American country. Because of lack of data, these region-specific estimates were not computed for Bolivia and Ecuador; nevertheless, evidence from Peruvian regions can inform regions with similar profiles in these countries.

Because diabetes is the outcome of physiological changes in glucose metabolism, and these changes occur over years, we assumed a 5-year time lag for the association between high BMI and diabetes. Therefore, for the analysis at the country level, the number of diabetes cases in 2014 attributable to high BMI were computed using BMI estimates in 2010. Also because of data availability, this time lag for the PAF estimates at the region level in Peru was set at 4 years, i.e. using BMI estimates in 2014 we computed the number of diabetes cases attributable to high BMI in 2017. Older or more recent nationally representative surveys in Peru were not available.

### 2.3 Data sources

For the analysis at the country level, estimates of exhaustive and mutually exclusive BMI categories in Bolivia, Ecuador and Peru were retrieved from the NCD-RisC (http://ncdrisc.org). This provided age-standardized prevalence estimates for men and women aged ≥20 years. The BMI estimates in the years 1980, 1990, 2000, 2005, 2010 and 2014 were used for descriptive purposes, though -as described above- BMI estimates in 2010 were used to compute the number of diabetes cases attributable to high BMI. BMI estimates were assessed with measured weight and height [12]. Diabetes estimates in 2014 were also retrieved from the NCD-RisC. These estimates were defined following consistent and comparable definitions and biomarkers: fasting plasma glucose ≥7.0 mmol/L, a history of diabetes (regardless of type 1 or 2) or use of insulin or oral hypoglycaemic drugs.

For the analysis at the region level in Peru, exhaustive and mutually exclusive BMI categories were extracted from the 2014 Demographic and Health Survey (DHS, ENDES in Spanish). BMI was based on measured weight and height of men and women aged ≥20 years. As the Peruvian DHS asked only for self-reported diagnosis of diabetes, we adjusted these self-reported diabetes prevalence estimates in each region in Peru to also account for unknown diabetes. We multiplied the self-reported estimates by the proportion of people living in urban and rural areas in each region times a correction factor for undiagnosed diabetes. The correction factor was derived from a population-based study conducted in three cities in Peru including urban and rural sites [17].

The relative risks quantifying the association between a five unit increase in BMI and diabetes risk were re-analysed in each of the two accessed cohorts independently [13], [14]. These risk estimates were pooled following a random-effects meta-analysis.

### 2.4 Statistical analysis

Benefiting from the detailed data sources, and assuming a 5-year time lag (4 years for region-specific analysis), we computed the proportion of diabetes cases attributable to high BMI using the following formula: [18], [19]

$$\mathrm{PAF}=\frac{\sum PiRRi-\sum P\text{'}iRRi}{\sum PiRRi}$$

where *Pi* is the actual distribution of BMI, i.e. the prevalence of each *i^th^* exhaustive and mutually exclusive BMI category; *P’i* is the prevalence in the alternative (ideal) scenario; and *RRi* is the adjusted relative risk of the association between high BMI and diabetes. To estimate the diabetes PAF attributable to high BMI (5-unit increase), the alternative optimal scenario was BMI <25 kg/m^2^. An equivalent formula was used when computing the PAF for BMI categories (25–29 [overweight], 30–34 [class I obesity], 35–39 [class II obesity], and ≥40 kg/m^2^ [class III obesity]), the alternative scenario was also BMI <25 kg/m^2^.

Using the computed PAF, both at the country and region level, we then estimated the number of diabetes cases attributable to high BMI. To do this, we multiplied the PAF computed for each country and region by the number of people with diabetes in each country and region. This operation gave the absolute number of diabetes cases attributable to high BMI in each country and region.

Uncertainty of our estimates were computed following a simulation approach wherein the uncertainty of the BMI and diabetes prevalence estimates, and the RRs, were propagated to the final estimates by taking 1000 random draws assuming a log-normal distribution informed by the mean and standard deviation of the prevalence estimates. Similarly, we propagated the uncertainty of the RRs with 1000 log-normal random draws, where the mean was the RR estimate and the standard deviation was computed from the standard error. We repeated the PAF and number of attributable diabetes cases calculations for each of these 1000 draws; thus, we had 1000 PAF results which represented the uncertainty distribution. Finally, the median of the 1000 draws was extracted as the main result, and the 95% uncertainty intervals (95% UI) corresponded to the 2.5 and 97.5 percentiles of the 1000 random draws.

### 2.5 Role of the funding source

The funder of the study had no role in study design, data collection/collation, data analysis, data interpretation, or writing of the report. RMC-L had full access to all the data in the study and had final responsibility for the decision to submit for publication. All authors approved the submitted version.

## 3 Results

### 3.1 Country level results – attributable fractions and absolute numbers

**Fig. 1:**
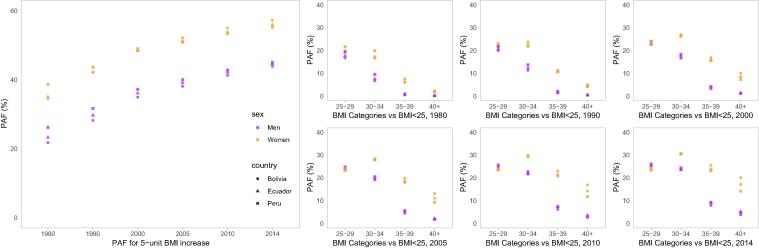
Population attributable fraction (PAF, %) of high body mass index (BMI) by sex and country across years of observation.

The proportion of diabetes cases attributable to each five-unit increase in BMI increased between 1980 and 2014, from an average of 29.9% to 50.3%, collectively in Bolivia, Ecuador and Peru. Over the study period, the fraction of diabetes attributable to high BMI across countries increased from 21.8–26.2% to 43.9–45.0% in men and from 34.6–38.6% to 55.2–57.2% in women (Fig. 1). The absolute increase was greatest for Bolivia, where the attributable fraction increased from 21.8% to 43.9% in men and from 34.6% to 57.2% in women. Conversely, the smallest increase was for Peru: from 26.2% to 45.0% in men and from 38.6% to 55.2% in women (Fig. 1).

Among the overweight and obesity categories, the greatest absolute increase was seen in class I obesity, for which the mean attributable fraction across countries more than doubled from 12.9% in 1980 to 27.2% in 2014. The second greatest increase was seen in class II obesity, increasing from 3.6% to 16.5%. Class III obesity also increased substantially, from 1.0% to 10.9%, representing the largest relative increase. The absolute increase was the least for overweight, which went from an average of 19.2% to 24.6% (Fig. 1). In the observation period, women have experienced a larger attributable fraction to BMI above 30 kg/m^2^; however, men have surpassed women in the overweight range (Fig. 1).

**Fig. 2:**
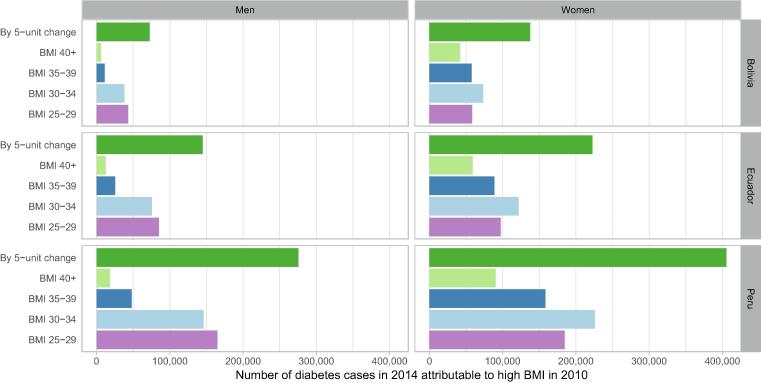
Absolute number of diabetes cases in 2014 attributable to high body mass index (BMI) in 2010 by sex and country.

In 2014 in Andean Latin-America, 1,258,313 diabetes cases were attributable to high BMI in 2010: 209,855 in Bolivia, 367,440 in Ecuador, 681,018 in Peru (Fig. 2). Consistently, a higher number of diabetes cases attributable to high BMI were observed in women than men, with the most in Peru: 405,547 in women vs 275,471 in men, followed by Ecuador (222,592 in women and 144,848 in men) and Bolivia (137,572 in women and 72,283 in men).

### 3.2 Region level estimates – attributable fractions and absolute numbers

**Fig. 3:**
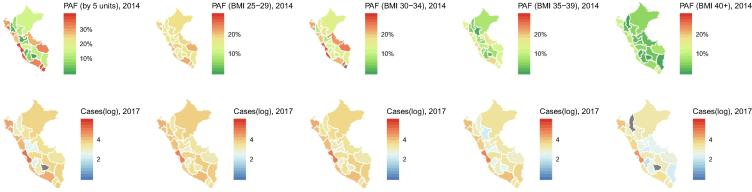
Population attributable fraction (PAF, %) of high body mass index (BMI) in 2014 by region in Peru and absolute number of diabetes cases (log scale) in 2017 attributable to high body mass index in 2014 by region in Peru.

We found substantial variation in the proportion of attributable cases by geography, whereby coastal regions in Peru had the largest burden of their diabetes cases attributable to high BMI (Fig. 3). These results signal a within country heterogeneous profile dominated by high-income coastal cities with large proportion of diabetes cases in 2017 attributable to high BMI in 2014. Lima, the highly-urbanized capital city of Peru, had the highest number of diabetes cases attributable to high BMI (147,575). On the other hand, Apurimac, an Andean city at ~5300 m above the sea level ranked last with zero cases.

Across Peruvian regions in 2014, women had a larger proportion of diabetes cases attributable to high BMI than men; likewise, the absolute number of attributable cases was larger in women than in men (Supplementary Fig. 1). In coastal high-income settings, the women-men ratio varied from 1.4 (Arequipa) to 3.7 (Ancash). This ratio in the Andes region ranged from 3.2 (Cusco) to 49.2 (Pasco). In the Amazon region these numbers were 1.8 (Ucayali) and 2.8 (Loreto), respectively. The widest range was found in the Andes regions, which could also be the case in similar regions in Bolivia and in the Andean regions of Ecuador.

In 2014, twelve (ten in the coast and two in the Amazon region) out of twenty-five regions, had diabetes burdens with larger attributable fractions due to class I obesity than to overweight. Conversely, in all Andean regions the attributable fraction was largest due to overweight. The attributable fraction due to class III obesity was ≥10% in six regions, all of which were in the Coast: Moquegua (14.4%), Callao (11.0%), Piura (11.0%), Lambayeque (10.5%), Lima (10.5%) and Tacna (10.3%). An attributable fraction due to class III obesity between 5% and 9% was found in eight regions (five in the coast, two in the Amazon and one in the Andes; Fig. 3).

### 3.3 Correlates of BMI related attributable fraction

**Fig. 4:**
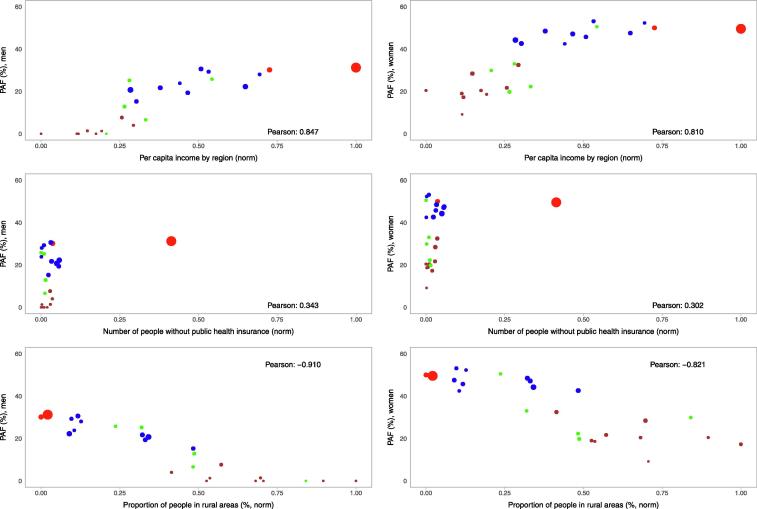
Population attributable fraction (PAF, %) for men and women by selected socio-demographic variables by region in Peru. Red colour represents Lima and Callao; blue colour represents the rest of the cities in the coast; brown colour represents cities in the Andes whereas green colour represents cities in the Amazon region. The size of the circles is proportional to the number of diabetes cases in 2017 attributable to high body mass index (BMI) in 2014, i.e., the larger the circle the more attributable cases there were. (For interpretation of the references to colour in this figure legend, the reader is referred to the web version of this article.)

We found a strong positive correlation between the proportion of diabetes cases attributable to high BMI and socio-economic variables at the region level (Fig. 4). Regions where the per capita income is high have larger proportion of attributable cases (Pearson correlation = 0.847 in men and 0.810 in women; Fig. 4). Similarly, regions where there are more people without health insurance, the proportion was larger (0.345 in men and 0.303 in women; Fig. 4). Conversely, there was a strong negative correlation with rurality; regions where more people live in rural areas had smaller proportion of attributable cases (−0.915 in men and −0.820 in women; Fig. 4).

## 4 Discussion

### 4.1 Main findings

The number and proportion of diabetes cases attributable to high BMI have increased in the last thirty years in Andean Latin-America, particularly affecting women and middle-aged individuals, with the greatest absolute increases seen in class I and class II obesity. In Andean Latin-America, 1,258,313 diabetes cases in 2014 were attributable to the prevalence of high BMI in 2010. Peruvian coastal regions and with high socio-economic status had a larger proportion of attributable cases and absolute number of cases compared to regions in the Andes and with sizable rural areas. The Amazon region showed heterogeneity with a few regions exhibiting high proportions and absolute estimates.

Our work provides timely estimates, strengthened by the granularity across key geographic targets for local and regional health authorities to plan interventions, set priorities and goals, optimally allocate intervention resources, and monitor programs. This work also provides evidence to monitor, guide and inform the WHO NCD Global Monitoring Framework, whereby countries -including Bolivia, Ecuador and Peru- have committed to a 0% increase in obesity and diabetes.

### 4.2 Public health relevance

We found a substantial increase in the burden of diabetes attributable to class I and class II obesity; this finding has relevant implications for the treatment and management of the increasing co-/multi-morbidity burden. In addition to subsequent risk of diabetes and cardiovascular outcomes, (extreme) high BMI is associated with other sequelae including several common cancers [20], fatty liver disease [21], worse lung function [22], and larger health economic burden [23], not only in comparison to normal weight people but also relative to overweight or less obese individuals. Globally, public health policies to date have failed to effectively tackle the obesity epidemic. However, evidence based, comprehensively implemented population policies have the potential to reduce the mean BMI in the whole population, thus decreasing the prevalence of overweight and possibly obesity. Dramatic changes would be needed to benefit those at the extreme of the distribution, i.e. class III obesity. Thus, these findings suggest that effectively managing the increasing diabetes epidemic and preventing further increases will require a combination of population-wide approaches along with interventions targeting those at highest risk, consistent with the Proportionate Universalism approach [24]. This therefore requires structural interventions to reduce the population’s risk, but additional targeted resources to those at highest risk and/or already severely obese.

Individuals at high risk of diabetes, pre-diabetes, or even with diabetes, could be targeted with intensive efforts to prevent, delay or revert their status. The Look AHEAD study [25], and more recently the DiRECT trial, have reported diabetes remission following intensive dietary interventions [26], with early results promising sustainability of effect after two years [27]. As we understand more about the pathophysiology of diabetes attributable to high BMI in specific populations, and the potential pathways for diabetes remission [28], this will guide the formulation of the most cost-effective risk stratification approaches for screening and to provide treatment as needed [29], [30]. Research in development, validation and implementation of screening tools should be promoted along with securing resources for treatment allocation overcoming socio-economic inequalities [31]. However, risk- and population-based interventions are needed to prevent or delay diabetes onset, as acknowledged by the PAHO [32]. This would require a paradigm change, whereby we transit from a curative model focused on treatment and prevention of complications, to a model in which primary prevention addressing essential medical and socio-economic risk factors prevails. In this line, population-based prevention strategies should include healthy food consumption [33], physical activity [34], [35], and environmental risk factors [36], [37], [38]. Strong political actions and commitment from different sectors will be needed to ensure structured multi-level interventions.

High BMI and diabetes have several shared risk factors including unhealthy diet and physical inactivity. Evidence-based health policies are available to tackle these shared risk factors and to prevent diabetes in Latin-America; the most effective interventions in the general population have been found to be those that addresses the obesogenic environment, fiscally optimising food choices and reformulating unhealthy foods [39], [40], [41]. Several efforts are underway in the Andean Latin-America region. Since 2014–2016, Ecuador and Bolivia have guidelines to improve the population nutritional profile and to prevent obesity, including a food labelling program using a traffic-light approach [42], [43], [44]. In Peru, a law to include octagons on foods labels signalling “high in…” but without the traffic-light colour pattern, was approved in 2017 and has been implemented since June 2019. Nonetheless, local experts have criticised the current guidelines arguing that the limits are too high to have a meaningful positive impact on population nutrition [45]. The effect of food labelling has not been extensively studied in low- and middle-income countries where consumers may have different profiles than their peers in high- income countries, where evidence about the positive effect of food labelling interventions is still inconclusive [41]. Because there is a positive association between ultra-processed foods and drinks sales and BMI at the population level [46], policies should look for the strongest interventions to reduce their purchasing and consumption.

Accounting for the biological factors behind the incremental trends in BMI and diabetes attributable to high BMI, early life programming could potentially explain these phenomena [47], [48], [49]. Changing rates of undernutrition could be an additional factor affecting the increase in BMI and diabetes prevalence in Bolivia and Ecuador. In 1975 the undernutrition prevalence in children/adolescents aged 5 to 19 years, i.e. people that in 2010 were 40 to 54 years old thus at high diabetes risk, was 14.4% in Bolivia, 15.0% in Ecuador and 12.8% in Peru [12]. Countries with the highest undernutrition rate 35 years ago -Bolivia and Ecuador- also had the fastest increase in mean BMI and diabetes prevalence; [2], [12] conversely, Peru which had the lowest undernutrition rate also exhibited the slowest increase in mean BMI and diabetes prevalence [2], [12].

Beyond biological determinants, social factors could have also influenced our findings and the increasing diabetes burden attributable to high BMI. For example, the rising BMI in rural areas [50] could explain the trends herein observed in the Peruvian Andes. Similarly, the countries we studied still face significant rural-to-urban migration, which could be linked to high obesity risk [51]. Even though people in high socio-economic status in many countries in Latin-America have healthier diets [52], the obesity prevalence difference between the highest and the lowest wealth index quintile has not changed much in Peruvian women (17% in 2005 vs 16% in 2017), whereas in Bolivian females the difference is slightly larger (14% in 1998 vs 19% in 2008); [53] the same pattern arises with education [53]. Because socio-economic level influences access to healthcare and treatment, a population-wide approach could benefit large populations (e.g., food taxes) or risk-based interventions could focus resources to those who most need them.

### 4.3 Results in context

Much of the evidence on comparative risk assessment analysis about cardio-metabolic risk factors and diseases has focused on hard endpoints such as non-fatal and fatal events [1], [54], including some examples from low and middle-income countries [55], [56], [57], [58] and Latin-America [59]. A few efforts, however, have studied diabetes cases instead. In China it has been estimated that high BMI is the leading attributable factor for diabetes, explaining almost half of the prevalence [60]. They also reported on several dietary factors that significantly contributed to the diabetes burden [60]. This study focuses upon BMI exclusively, without consideration of dietary components due to data availability; however, it is likely that such factors might also play a relevant role in the increasing diabetes burden in Andean Latin-America [61]. Similarly, physical (in)activity is an important determinant, but data on this health-related lifestyle is even scarcer. Nevertheless, available evidence already suggests that Latin-America and the Caribbean is a region with high prevalence of insufficient physical activity [62], rendering it as an important risk factor for public health and policy attention because physical activity could prevent or delay diabetes [63].

At the country and region level we observed higher PAF estimates for women than for men. We used the same relative risks across genders, thus these differences could be due to higher mean BMI and diabetes prevalence estimates in women than in men; robust evidence supports this argument [2], [12]. Similarly, regarding the region level analysis, higher BMI and diabetes incidence have been observed in women than in men [13], [64]. This female disadvantage has also been reported when looking at hard endpoints such as mortality, where higher proportions [55], [57] and more attributable deaths [58], [59] have been reported in women than in men.

### 4.4 Future needs

Our findings suggest there is a considerable number of diabetes cases attributable to high BMI across regions in Peru as well as in Bolivia, Ecuador and Peru. This highlights the need for a change in current paradigm, from one in which treatment is the priority to one in which primary prevention prevails with a strong focus on cardio-metabolic [2], [12], lifestyles [33], [34], [35], socio-economic and environmental risk factors [53], [36], [37], [38]. Political commitment and funding is needed to support specific actions to strengthen primary care [65]. This could include a focus on health outcomes of patients such as weight control or reduction, set a baseline profile, establish goals and monitor frequently, and incentive systems for goal achievement to improve the patients’ health.

From a research and data availability perspective, comparable, consistent and long-term evidence is needed to understand changes in key determinants including, but not limited to: diet and physical activity profiles, food security, and socio-economic inequalities and determinants. This evidence would inform interventions, policies and monitoring programs. Furthermore, this information is needed with the greatest granularity possible, moving beyond country-wide estimates.

### 4.5 Strengths and limitations

This work has benefited from national and international robust, consistent and comparable data sources. The results accounted for a time lag between the exposure and outcome. Moreover, we used local RRs estimating the association between high BMI and diabetes which should better inform the epidemiological scenario in the studied countries and regions than RRs from international populations with different health/epidemiological profiles. Nonetheless, there are also some limitations. First, the relative risks were computed from population-based studies including people aged ≥30 years. Therefore, future efforts should aim to include estimates for younger people. Second, the relative risks were computed from studies in Peru including a wide range of socio-economic, geographic and health profiles, though people from the Amazon region were not studied. However, other prospective cohort study in Peru which included people from the Amazon region reported very similar results to our cohorts [15], supporting the argument that our risk estimates could inform the Amazon region as well. Third, the diabetes prevalence estimates could not separate type 1 and type 2 diabetes. Because at the population level the frequency of type 1 diabetes is rather small, this caveat would not affect the results and conclusions. Fourth, because of data availability, we could not compute the country-level estimates stratified by rural-urban location. Nevertheless, the results are still informative for these countries, and this work could spark interest to produce consistent and comparable information by urban and rural location. Of note, the excess of BMI in urban versus rural areas in these countries ranges from 0.93 kg/m^2^ in Ecuadorian women to 2.50 kg/m^2^ in Peruvian men [50].

## 5 Conclusion

A substantial and increasing number of diabetes cases are attributable to high BMI in the Andean Latin-American region. The largest burden of diabetes attributable to high BMI was for woman, urban areas and in high-income settings whereas places with rural environments show fewer attributable cases. Andean Latin-America public health policies should pursue a dual approach to this growing epidemic; implement population-based interventions to reduce high BMI, alongside targeted clinical programmes for those who are already obese and/or at highest risk of developing diabetes.

## 6 Research in Context

### 6.1 Evidence before this study

We searched MEDLINE through PubMed on September 2nd, 2019 using the search formula: “comparative risk assessment“ AND (”BMI“ OR ”body mass index“) AND ”diabetes“ AND (”Bolivia“ OR ”Ecuador“ OR ”Peru“). A second search strategy using the terms: (”BMI“ OR ”body mass index“) AND ”diabetes“ AND (”Bolivia“ OR ”Ecuador“ OR ”Peru“), retrieved 75 articles but none reported on population attributable fractions or absolute number of attributable cases at the country/region level. The findings from these searches reflect the lack of comprehensive, comparable and consistent population health metrics to inform policies and interventions to reduce the burden of high body mass index (BMI) on diabetes in Andean Latin-America (Bolivia, Ecuador and Peru).

### 6.2 Added value of this study

Using the most comprehensive available estimates of diabetes and BMI categories prevalence estimates, along with risk estimates computed from population-based cohorts in Peru, we showed that approximately 1.3 million diabetes cases were attributable to high BMI in Andean Latin-America. Interestingly, and not quantified ever before, we have shown that the attributable fraction to class I, class II and class III obesity has increased more than that of overweight, suggesting an overwhelming burden of the extreme of the BMI distribution. The region-level findings in Peru clearly depicted a heterogeneous geographic distribution, with high-income regions exhibiting larger attributable fractions than low- and middle-income regions.

### 6.3 Implications of all the available evidence

As the prevalence of both high BMI and diabetes continues to increase in Andean Latin-America, a multi-faceted approach to prevention and management of high BMI and diabetes risk is required. Health systems, alongside their ambition to achieve universal health coverage, should invest in preventative measures with the dual aim of reducing the population’s BMI hence diabetes risk, and additionally targeting those at highest and most urgent risk of developing diabetes such as those in class II or III obesity.

## 7 Authors’ contribution

RMC-L conceived the idea with JP-S, EWG and AB-O. RMC-L conducted the analysis with support from JP-S and AB-O. RMC-L drafted the first version of the manuscript. All authors provided relevant scientific contribution. All authors approved the submitted version.

## Funding

Rodrigo M. Carrillo-Larco has been supported by Strategic Award, Wellcome Trust-Imperial College Centre for Global Health Research (100693/Z/12/Z), and Imperial College London Wellcome Trust Institutional Strategic Support Fund [Global Health Clinical Research Training Fellowship] (294834/Z/16/Z ISSF ICL). Rodrigo M Carrillo-Larco is supported by a Wellcome Trust International Training Fellowship (214185/Z/18/Z).

## Declaration of Competing Interest

The authors declare that they have no known competing financial interests or personal relationships that could have appeared to influence the work reported in this paper.
