# Supplementary material for: The Andean Latin-American burden of diabetes attributable to high body mass index: A comparative risk assessment
Source: Diabetes Res Clin Pract. 2020 Feb;160:107978. doi: 10.1016/j.diabres.2019.107978 (PMC7042885; doi:10.1016/j.diabres.2019.107978)

**Supplementary Material**

**The Andean Latin American burden of diabetes attributable to high body mass index: A comparative risk assessment**

**Corresponding author:**

Rodrigo M. Carrillo-Larco, MD

Department of Epidemiology and Biostatistics

School of Public Health

Imperial College London, London, UK

[rcarrill@ic.ac.uk](mailto:rcarrill@ic.ac.uk)

**Contents**

[Epidemiological profile comparison among Bolivia, Ecuador and Peru 3](#_Toc18352917)

[Expanded methods 4](#_Toc18352918)

[1. Data sources 4](#_Toc18352919)

[2. Adjusting self-reported diabetes prevalence estimates at the region level in Peru 5](#_Toc18352920)

[3. Age standardization – Peruvian DHS estimates 6](#_Toc18352921)

[4. Time lag 7](#_Toc18352922)

[5. Population attributable fraction (PAF) 7](#_Toc18352923)

[6. Number of attributable cases 8](#_Toc18352924)

[7. Uncertainty 8](#_Toc18352925)

[8. Socio-economic co-variables 8](#_Toc18352926)

[References 9](#_Toc18352927)

[Obesity and diabetes prevalences by country and sex 10](#_Toc18352928)

[Population attributable fraction by country and sex 12](#_Toc18352929)

[Population attributable fraction cases by regions in Peru 16](#_Toc18352930)

[Population attributable fraction and number of attributable cases by region in Peru in men 18](#_Toc18352931)

[Population attributable fraction and number of attributable cases by region in Peru in women 20](#_Toc18352932)

[Supplementary Figure 1: Population attributable fraction (PAF, %) and absolute number of diabetes cases in 2017 attributable to high body mass index (BMI) in 2014 in women versus men by region in Peru. 22](#_Toc18352933)

# **Epidemiological profile comparison among Bolivia, Ecuador and Peru**

Overall, these are middle-income countries where death rates for major non-communicable diseases, as well as prevalence estimates of diabetes and obesity are comparable among them. The following estimates support the comparisons among countries and using Peruvian regions as a case study to provide evidence for similar regions in Bolivia and Ecuador.

|  | **Bolivia** | **Ecuador** | **Peru** |
| --- | --- | --- | --- |
| Population (2017) | 11.05 millions | 16.62 millions | 32.17 millions |
| Income level ^a^ | Lower middle income | Upper middle income | Upper middle income |
| GDP ($), 2017 ^a^ | 37,509 | 104,296 | 211,389 |
| Ischaemic heart disease death rates ^b^ | 13.7% (12.5%-15.1%) of total deaths | 11.3% (10.6%-12.0%) of total deaths | 11.7% (11.1%-12.5%) of total deaths |
| Stroke death rates ^b^ | 6.2% (5.4%-7.0%) of total deaths | 5.4% (5.0%-5.8%) of total deaths | 4.6% (4.2%-4.9%) of total deaths |
| Diabetes death rates ^b^ | 3.9% (3.4%-4.5%) of total deaths | 5.4% (5.1%-5.7%) of total deaths | 3.0% (2.8%-3.1%) of total deaths |
| Mean BMI, women in 2016 ^c^ | 27.9 (26.5-29.3) Kg/m^2^ | 28.1 (27.0-29.2) Kg/m^2^ | 27.3 (26.8-27.8) Kg/m^2^ |
| Mean BMI, men in 2016 ^c^ | 25.4 (23.7-27.1) Kg/m^2^ | 26.8 (25.8-27.7) Kg/m^2^ | 26.4 (25.9-26.9) Kg/m^2^ |
| Obesity prevalence, women in 2016 ^c^ | 26.6% (19.2%-34.4%) | 25.6% (18.6%-33.4%) | 25.1% (20.4%-30.2%) |
| Obesity prevalence, men in 2016 ^c^ | 15.1% (8.8%-22.8%) | 15.4% (9.6%-22.5%) | 15.8% (11.4%-20.5%) |
| Diabetes prevalence, women 2014 ^c^ | 8.9% (4.1%-15.1%) | 8.5% (3.7%-14.9%) | 8.1% (3.8%-13.7%) |
| Diabetes prevalence, men 2014 ^c^ | 7.0% (2.9%-12.4%) | 7.5% (3.1%-13-2%) | 7.2% (3.2%-12.6%) |

a Estimates provided by the World Bank, available at <https://data.worldbank.org/country>

b Deaths rates are for both men and women and all ages in 2016; estimates from the Global Burden of Disease Study and can be found at <https://gbd2016.healthdata.org/gbd-compare/>

c BMI and obesity estimates are age-standardized for people ≥20 years, whereas diabetes estimates are age-standardised for adults ≥18 years; estimates come from the NCD-RisC and can be found at <http://ncdrisc.org/data-visualisations.html>

# **Expanded methods**

The following pages provide in-depth details of the analytical approach. The R code used for this work will be available upon publication; please do not hesitate to contact the corresponding author for requests. The overall proceedings are shown in the following figure while in-depth details follow.

**BMI**

**Relative risks**

**PAF** (formula)

**Diabetes prevalence**

**Number of attributable cases**

**DHS**

**NCD-RisC**

**Cohorts**

**DHS**

**NCD-RisC**

Results at the country and region level are presented overall as well as sex-stratified.

## **Data sources**

For a comparative risk assessment analysis three components are needed:

1. Distribution of the risk factor of interest (in this case BMI)
2. Distribution of the outcome of interest (in this case diabetes prevalence)
3. Relative risk of the association between the risk factor (i) and the outcome (ii) of interest

We used open and accessible data sources to inform the metrics of interest at the country (Bolivia, Ecuador and Peru) and region level in Peru (25 regions).

| **Level** | **BMI** | **Diabetes** | **Relative Risk** |
| --- | --- | --- | --- |
| Country  (Bolivia, Ecuador and Peru) | NCD-RisC prevalence of exhaustive and mutually exclusive BMI categories in each country: BMI <18.5, [18.5-20[, [20-24.9[, [25-29.9[, [30-34.9[, [35-40[ and ≥40. These estimates can be downloaded from: <http://ncdrisc.org/data-downloads.html> | Prevalence estimates and the number of people with diabetes in each country were downloaded from the NCD-RisC: <http://ncdrisc.org/data-downloads.html> | We used two population-based cohorts in Peru.^1-3^ These were re-analysed to estimate the relative risk from 5-unit increase in BMI to diabetes. In other words, BMI in a continuous scale divided by 5 was the exposure variable, whereas new diabetes cases was the outcome. These risk estimates were computed for men and women ≥35 years old. |
| Peruvian regions  (25 regions) | The Peruvian DHS surveys were re-analysed to extract exhaustive and mutually exclusive BMI categories in each of the 25 regions of Peru: BMI <20, [20-24.9[, [25-29.9[, [30-34.9[, [35-40[ and ≥40. These surveys can be downloaded from: <http://iinei.inei.gob.pe/microdatos/> | The Peruvian DHS surveys were re-analysed to extract the prevalence of self-reported diabetes in each of the 25 regions of Peru. These surveys can be downloaded from: <http://iinei.inei.gob.pe/microdatos/> |  |

We used the prevalence of BMI exhaustive and mutually exclusive categories from the NCD-RisC (<http://ncdrisc.org/index.html>) to inform metrics at the country level (Bolivia, Ecuador and Peru). Similarly, we used the prevalence of BMI exhaustive and mutually exclusive categories from the Peruvian DHS surveys which can be downloaded from the Peruvian National Institute of Statistics and Informatics (<https://www.inei.gob.pe/>).

In a similar way, diabetes prevalence estimate at the country level were downloaded from the NCD-RisC for each country of interest. However, the Peruvian DHS surveys, used to inform metrics at the region level in Peru, only collected self-reported diabetes diagnosis. These self-reported estimates were adjusted to account for unknown diabetes (details follow). Therefore, at the country (NCD-RisC) and at the region level (DHS adjusted estimates), consistent and comparable diabetes prevalence estimates were available.

The relative risk between BMI and diabetes were extracted from two population-based cohorts in Peru.^1-3^ The relative risks were computed using BMI as a continuous variable divided by 5 as the exposure of interest, whereas new diabetes cases was the outcome in the Poison regression model. These risk estimates were adjusted by relevant confounders according to data availability in each cohort, including sex, age, socio-economic status and education. These cohort studies targeted middle-aged adults (≥35 years). The relative risks of the cohorts were pooled with a random-effects meta-analysis. Because the relative risk estimates were similar across age groups (35-49, 50-64 and ≥65), for simplicity we used one overall relative risk, i.e. not sex- or age-group-specific. The relative risk estimates were:

| Source | Population | Coef. (SE) | p | RR (95% CI) |
| --- | --- | --- | --- | --- |
| CRONICAS^4^ | **All** | **0.58815 (0.09069)** | <0.001 | 1.80 (1.62-1.98) |
|  | 35-49 years | 0.59729 (0.16573) | <0.001 | 1.82 (1.49-2.14) |
|  | 50-64 years | 0.61369 (0.12826) | <0.001 | 1.85 (1.60-2.10) |
|  | ≥65 years | 0.48767 (0.21846) | <0.050 | 1.63 (1.20-2.06) |
|  |  |  |  |  |
| Source | Population | Coef. (SE) | p | RR (95% CI) |
| PERU MIGRANT^2^ | **All** | **0.714690 (0.13859)** | <0.001 | 2.04 (1.77-2.32) |
|  | 35-49 years | 0.525410 (0.19755) | <0.010 | 1.69 (1.30-2.08) |
|  | 50-64 years | 0.842464 (0.21817) | <0.001 | 2.32 (1.90-2.75) |
|  | ≥65 years | 0.487670 (0.21846) | <0.050 | 1.63 (1.20-2.06) |

Estimates in bold were used in the analysis.

These pooled relative risks were used to compute the population attributable fractions. Two potential limitations of these pooled relative risks are worth mentioning: i) none of these studies included people from the Amazon region in Peru, thus population attributable fraction estimates for this region should be interpreted in light of this pitfall; and ii) the pooled relatives risks were informed by relative risks estimated in people aged ≥35 years, yet the population attributable fractions included younger people (<35 years). Because the magnitude of the relative risk could be slightly smaller in people younger than 35 years old, the population attributable fraction estimates for these age groups should be interpreted along with this potential limitation. Despite these potential limitations, this work provides evidence to inform policies and priorities to reduce the obesity and diabetes burden in Bolivia, Ecuador and Peru, where currently evidence-based policies seriously lack.

## **Adjusting self-reported diabetes prevalence estimates at the region level in Peru**

The Peruvian DHS surveys do not collect any biomarkers to test for glucose; i.e., these surveys only ask if the participant has been diagnosed with diabetes or high blood glucose/sugar. Therefore, these self-reported estimates do not reflect the real prevalence of diabetes in Peru, because they do not account for unaware/undiagnosed diabetes. We re-analysed the baseline assessment of the CRONICAS Cohort Study,^4^ a population based-cohort study conducted in three different cities in Peru to compute the following:

| Metric | Lima (%) [urban] | Tumbes (%) [semi-urban] | Urban Puno (%)  [urban] | Rural Puno (%)  [rural] | Comment |
| --- | --- | --- | --- | --- | --- |
| a)Diabetes prevalence | 4.46 | 8.54 | 6.00 | 2.95 | overall diabetes prevalence: self-reported and biomarkers |
| b)Self-reported diabetes | 2.62 | 4.55 | 2.62 | 0.57 | self-reported diabetes prevalence |
| a/b | 1.70 | 1.88 | 2.29 | 5.18 | proportion due to new cases diagnosed with biomarkers |

With these prevalence estimates by urban and rural sites, we computed a weight factor specific for urban and rural settings; these weights multiplied the self-reported diabetes prevalence estimates obtained from the Peruvian DHS to compute a diabetes prevalence estimate accounting for undiagnosed diabetes as well, i.e., not only self-reported or aware diabetes prevalence estimates. The weight factor for urban areas was 1.96 (~100/51.1), the mean among 1.70 (Lima), 1.88 (Tumbes) and 2.29 (urban Puno); on the other hand, the weight factor for rural areas was 5.13. We used these urban/rural-specific weights, along with the proportion of people in urban and rural sites in each region in Peru, to adjust the self-reported diabetes prevalence estimates as follow:

$$Diabetes Prevalence in region 1=self reported diabetes in Region 1 x proportion of people in uban areas in region 1 x urban weight + self reported diabetes in Region 1 x proportion of people in rural areas in region 1 x rural weight$$

The above formula was computed for each of the 25 Peruvian regions, using the corresponding proportions of people in urban or rural areas; these proportions were retrieved from the 2017 Peruvian National Census (http://censos2017.inei.gob.pe/redatam/). This approach was used to compute the diabetes prevalence overall (including both men and women) and sex-specific.

## **Age standardization – Peruvian DHS estimates**

The prevalence estimates of all BMI categories as well as the prevalence of self-reported diabetes prevalence, variables originally extracted from the Peruvian DHS, were age standardized according to the population in the 2017 Peruvian National Census. The prevalence estimates were multiplied by the derived ratio: overall and sex-specific.

| All | ≥20 years | 19,174,810 | All ages | 29,347,115 | Ratio = 0.6533797 |
| --- | --- | --- | --- | --- | --- |
| Men | ≥20 years | 9,279,683 | All ages | 14,433,419 | Ratio = 0.6429303 |
| Women | ≥20 years | 9,895,127 | All ages | 14,913,969 | Ratio = 0.6634926 |

Ratio refers to ≥20 years divided by all ages (i.e., 19,174,810 / 29,347,115 = 0.6533797).

## **Time lag**

Assuming BMI is a casual factor for diabetes onset, there would be a time lag between these two conditions, i.e., some years are needed for someone with high BMI to develop diabetes. We assumed a time lag of 5 years between high BMI and diabetes onset. Therefore, to compute the number of diabetes cases attributable to high BMI at the country level, we assumed a 5-year time lag: BMI distribution in 2010 to compute the number of diabetes cases in 2014. Because of data availability, to compute the number of diabetes cases due to high BMI at the region level in Peru, we assumed ~4-year time lag: BMI distribution in 2014 to compute the number of diabetes cases in 2017.

| Location | Exposure | Outcome |
| --- | --- | --- |
| Country level analysis | BMI distribution in 2010 | Diabetes prevalence estimates in 2014 |
| Region level analysis in Peru | BMI distribution in 2014 | Diabetes prevalence estimates in 2017 |
| Rationale | There should be a time lag between being exposed to high BMI levels and diabetes onset; in other words, the casual effect of high BMI on diabetes onset occurs in years, not immediately after. | |

## **Population attributable fraction (PAF)**

The PAF attributable to high BMI was estimates following standard methods and this formula:^5, 6^

$$PAF= \frac{\sum PiRRi- \sum P'iRRi}{\sum PiRRi}$$

Where *Pi* is the actual distribution of BMI, i.e., the prevalence of each *i^th^* BMI category; *P’i* is the prevalence in the alternative (ideal) scenario; and *RRi* is the adjusted relative risk of high BMI associated with diabetes. Please, for further details on how to compute this formula across BMI categories, refer to the available analysis code. An example is provided below:

pafbmi.dt$paf.by.5 <- ((pafbmi.dt$p_bmi_20 * 1) +

(pafbmi.dt$p_bmi_20.25 * 1) +

(pafbmi.dt$p_bmi_25.30 * pafbmi.dt$bmi.rr.exp) +

(pafbmi.dt$p_bmi_30.35 * pafbmi.dt$bmi.rr.exp^2) +

(pafbmi.dt$p_bmi_35.40 * pafbmi.dt$bmi.rr.exp^3) +

(pafbmi.dt$p_bmi_40 * pafbmi.dt$bmi.rr.exp^4) - 1) /

((pafbmi.dt$p_bmi_20 * 1) +

(pafbmi.dt$p_bmi_20.25 * 1) +

(pafbmi.dt$p_bmi_25.30 * pafbmi.dt$bmi.rr.exp) +

(pafbmi.dt$p_bmi_30.35 * pafbmi.dt$bmi.rr.exp^2) +

(pafbmi.dt$p_bmi_35.40 * pafbmi.dt$bmi.rr.exp^3) +

(pafbmi.dt$p_bmi_40 * pafbmi.dt$bmi.rr.exp^4))

This example shows that the prevalence of each BMI category is multiplied by the relative risk; also, the relative risk is elevated to the power of 1, 2, 3 or 4 for BMI categories above the threshold set as ideal or optimal, i.e., for all BMI categories above 25. For categories below this threshold, these are multiplied by 1 and not by the relative risk because these BMI categories would not be expected to increase the risk of diabetes.

We also estimated the PAF for BMI categories (25-29, 30-34, 35-39 and ≥40) using a simplified form of the formula shown above. As explained before, the counterfactual ideal scenario would be BMI <25 kg/m^2^. For descriptive purposes, these PAF estimates were computed for all years of observation (1980, 1990, 2000, 2005, 2010, 2014), though again, the number of attributable cases were only assessed with 2010 PAF (and diabetes prevalence estimates in 2014).

$$PAF= \frac{Pi x (RR-1)}{Pi x \left( RR-1 \right)+1}$$

The corresponding code looks like:

pafbmi.dt.2010$paf.2010.vs.25_30 <-

(pafbmi.dt.2010$p_bmi_25.30 * (pafbmi.dt.2010$bmi.rr.exp - 1)) /

(pafbmi.dt.2010$p_bmi_25.30 * (pafbmi.dt.2010$bmi.rr.exp - 1) + 1)

[…]

pafbmi.dt.2010$paf.2010.vs.40 <-

(pafbmi.dt.2010$p_bmi_40 * (pafbmi.dt.2010$bmi.rr.exp^4 - 1)) /

(pafbmi.dt.2010$p_bmi_40 * (pafbmi.dt.2010$bmi.rr.exp^4 - 1) + 1)

The PAF underlying concept is: a proportional reduction of the outcome of interest that would occur if the exposure to the risk factor was reduced to an alternative (ideal) scenario.^5^ Regarding this work, this would mean: a proportional reduction in the cases of diabetes that would occur if BMI was reduced to an alternative healthy (ideal) scenario where the population BMI is below 25 Kg/m^2^.

## **Number of attributable cases**

The PAF, as computed above, multiplied by the number (national or regional) of diabetes cases, will provide the number of diabetes cases that can be attributable to high BMI (the exposure of interest). Regarding the country-level analysis the proposed interpretation would be: in the counterfactual scenario in which the BMI distribution is within normal ranges, X cases of diabetes would be avoided; alternatively, in the current BMI distribution, X diabetes cases are attributable to high BMI. The same interpretations could be drawn from the region-level analysis.

## **Uncertainty**

To estimate uncertainty intervals, we followed a simulation approach throughout each component of the PAF formula. For example, at the country level, the prevalence estimates were replicated 18,000 times using the mean and the standard deviation. Likewise, 18,000 replications of the relative risks were drawn using the relative risk as the mean and the standard deviation (standard error to the power of 2). At the country level we made estimates for three countries and six years, namely eighteen analysis units (country-year); the reason to compute 18,000 replications was to have 1,000 replications for each analysis unit (country-year). Regarding the region-level analysis, there were 25,000 replications, i.e., one for each region-year. The full set of replications was used to conduct the analysis; however, the reported results correspond to the median and the 95% credible interval to the 2.5% percentile and the 97.5% percentile of the replication distributions.

## **Socio-economic co-variables**

Three socio-economic variables were used for correlation purposes at the region level in Peru: per-capita monthly income by region in Peru in 2017; people living in rural areas by region in Peru in 2017; people with and without public health insurance in 2017 by region in Peru. The first two variables were extracted from the 2017 Peruvian National Census, while the last one from the Peruvian 2017 DHS.

# **References**

1. Bernabe-Ortiz A, Carrillo-Larco RM, Gilman RH, et al. Geographical variation in the progression of type 2 diabetes in Peru: The CRONICAS Cohort Study. *Diabetes research and clinical practice* 2016; **121**: 135-45.

2. Carrillo-Larco RM, Ruiz-Alejos A, Bernabe-Ortiz A, Gilman RH, Smeeth L, Miranda JJ. Cohort Profile: The PERU MIGRANT Study-A prospective cohort study of rural dwellers, urban dwellers and rural-to-urban migrants in Peru. *International journal of epidemiology* 2017; **46**: 1752-f.

3. Ruiz-Alejos A, Carrillo-Larco RM, Miranda JJ, et al. Addressing the impact of urban exposure on the incidence of type 2 diabetes mellitus: The PERU MIGRANT Study. *Scientific reports* 2018; **8**: 5512.

4. Miranda JJ, Bernabe-Ortiz A, Smeeth L, Gilman RH, Checkley W. Addressing geographical variation in the progression of non-communicable diseases in Peru: the CRONICAS cohort study protocol. *BMJ open* 2012; **2**: e000610.

5. Ezzati M, Lopez AD, Rodgers A, Vander Hoorn S, Murray CJ. Selected major risk factors and global and regional burden of disease. *Lancet (London, England)* 2002; **360**: 1347-60.

6. Murray CJ, Ezzati M, Lopez AD, Rodgers A, Vander Hoorn S. Comparative quantification of health risks conceptual framework and methodological issues. *Population health metrics* 2003; **1**: 1.

# **Obesity and diabetes prevalences by country and sex**

| Country | Year | Sex | P(<18.5) | P[18.5-20[ | P[20-25[ | P[25-30[ | P[30-35[ | P[35-40[ | P(40+) | P(Diabetes) |
| --- | --- | --- | --- | --- | --- | --- | --- | --- | --- | --- |
| Bolivia | 1980 | Men | 0.053046 | 0.109335 | 0.579625 | 0.229013 | 0.028113 | 0.000795 | 7.43E-05 |  |
| Bolivia | 1980 | Women | 0.040302 | 0.082631 | 0.506597 | 0.279168 | 0.078135 | 0.011415 | 0.001752 |  |
| Bolivia | 1990 | Men | 0.036566 | 0.081686 | 0.544938 | 0.28396 | 0.050247 | 0.002317 | 0.000285 |  |
| Bolivia | 1990 | Women | 0.026825 | 0.060464 | 0.461146 | 0.315874 | 0.109532 | 0.021661 | 0.004499 |  |
| Bolivia | 2000 | Men | 0.024291 | 0.059665 | 0.497172 | 0.333336 | 0.078778 | 0.005747 | 0.001011 |  |
| Bolivia | 2000 | Women | 0.018974 | 0.046266 | 0.411943 | 0.337744 | 0.139405 | 0.035978 | 0.009691 |  |
| Bolivia | 2005 | Men | 0.019779 | 0.051206 | 0.472165 | 0.353438 | 0.093356 | 0.008317 | 0.001739 |  |
| Bolivia | 2005 | Women | 0.016504 | 0.04149 | 0.3893 | 0.343499 | 0.151997 | 0.043996 | 0.013214 |  |
| Bolivia | 2010 | Men | 0.01618 | 0.043973 | 0.445596 | 0.370924 | 0.108608 | 0.011791 | 0.002928 |  |
| Bolivia | 2010 | Women | 0.014612 | 0.037637 | 0.366679 | 0.346861 | 0.163648 | 0.052933 | 0.017629 |  |
| Bolivia | 2014 | Men | 0.013894 | 0.039021 | 0.423398 | 0.382587 | 0.12126 | 0.015428 | 0.004412 | 0.069502 |
| Bolivia | 2014 | Women | 0.013424 | 0.035076 | 0.348711 | 0.347823 | 0.172348 | 0.06072 | 0.021898 | 0.08948 |
| Ecuador | 1980 | Men | 0.036034 | 0.099514 | 0.587765 | 0.2434 | 0.032042 | 0.001145 | 0.0001 |  |
| Ecuador | 1980 | Women | 0.03663 | 0.079516 | 0.504973 | 0.283709 | 0.08233 | 0.011271 | 0.001571 |  |
| Ecuador | 1990 | Men | 0.023907 | 0.073696 | 0.54794 | 0.295841 | 0.055152 | 0.003119 | 0.000346 |  |
| Ecuador | 1990 | Women | 0.024688 | 0.058697 | 0.460886 | 0.318366 | 0.112737 | 0.020736 | 0.003891 |  |
| Ecuador | 2000 | Men | 0.015582 | 0.054075 | 0.498442 | 0.340674 | 0.082905 | 0.007192 | 0.00113 |  |
| Ecuador | 2000 | Women | 0.017719 | 0.045577 | 0.414698 | 0.339543 | 0.140973 | 0.033423 | 0.008066 |  |
| Ecuador | 2005 | Men | 0.012618 | 0.046436 | 0.472539 | 0.359152 | 0.09709 | 0.010232 | 0.001933 |  |
| Ecuador | 2005 | Women | 0.015428 | 0.040936 | 0.392773 | 0.345661 | 0.153558 | 0.040707 | 0.010938 |  |
| Ecuador | 2010 | Men | 0.010439 | 0.040327 | 0.446741 | 0.374141 | 0.11101 | 0.014114 | 0.003229 |  |
| Ecuador | 2010 | Women | 0.013759 | 0.037322 | 0.371784 | 0.349265 | 0.164822 | 0.048556 | 0.014491 |  |
| Ecuador | 2014 | Men | 0.009091 | 0.036201 | 0.425673 | 0.383793 | 0.122315 | 0.018079 | 0.004849 | 0.074656 |
| Ecuador | 2014 | Women | 0.012704 | 0.034903 | 0.355115 | 0.350611 | 0.173181 | 0.055456 | 0.018031 | 0.084554 |
| Peru | 1980 | Men | 0.036838 | 0.086925 | 0.56141 | 0.271724 | 0.041415 | 0.001584 | 0.000103 |  |
| Peru | 1980 | Women | 0.03371 | 0.063236 | 0.473331 | 0.315415 | 0.098112 | 0.014272 | 0.001925 |  |
| Peru | 1990 | Men | 0.024856 | 0.066147 | 0.524982 | 0.317144 | 0.062866 | 0.003673 | 0.000332 |  |
| Peru | 1990 | Women | 0.024337 | 0.050106 | 0.438158 | 0.339736 | 0.121774 | 0.022118 | 0.003771 |  |
| Peru | 2000 | Men | 0.016379 | 0.049416 | 0.479916 | 0.357599 | 0.088059 | 0.007638 | 0.000992 |  |
| Peru | 2000 | Women | 0.018549 | 0.041337 | 0.40076 | 0.355328 | 0.144644 | 0.032549 | 0.006835 |  |
| Peru | 2005 | Men | 0.013179 | 0.042337 | 0.454909 | 0.375709 | 0.101731 | 0.010527 | 0.001608 |  |
| Peru | 2005 | Women | 0.016486 | 0.038027 | 0.381411 | 0.360196 | 0.155868 | 0.039037 | 0.008975 |  |
| Peru | 2010 | Men | 0.010705 | 0.036274 | 0.428718 | 0.391642 | 0.115915 | 0.014224 | 0.002521 |  |
| Peru | 2010 | Women | 0.014922 | 0.035385 | 0.361584 | 0.363273 | 0.166597 | 0.04652 | 0.01172 |  |
| Peru | 2014 | Men | 0.009169 | 0.032112 | 0.407395 | 0.402318 | 0.12748 | 0.017951 | 0.003575 | 0.072326 |
| Peru | 2014 | Women | 0.01396 | 0.033634 | 0.345641 | 0.36438 | 0.174715 | 0.053203 | 0.014467 | 0.080752 |

Information available at: <http://ncdrisc.org/index.html>

NCD Risk Factor Collaboration (NCD-RisC). Worldwide trends in body-mass index, underweight, overweight, and obesity from 1975 to 2016: a pooled analysis of 2416 population-based measurement studies in 128·9 million children, adolescents, and adults. Lancet. 2017;390(10113):2627-2642.

# **Population attributable fraction by country and sex**

The absolute number of attributable cases of diabetes in 2014 can be computed multiplying the PAF with the number of people with diabetes in 2014. For example, the absolute number of diabetes cases in 2014 attributable to high body mass index (BMI) in 2010 in women in Bolivia is 0.413 x 175227 = 72,283. The PAF were computed for a 5-unit change in BMI and for different BMI ranges (e.g., 25-29, 30-35, 35-40, 40+).

| Country | Sex | PAF in 1980 | PAF in 1980, Lower Limit | PAF in 1980, Upper Limit | PAF in 1990 | PAF in 1990, Lower Limit | PAF in 1990, Upper Limit | PAF in 2000 | PAF in 2000, Lower Limit | PAF in 2000, Upper Limit | PAF in 2005 | PAF in 2005, Lower Limit | PAF in 2005, Upper Limit | PAF in 2010 | PAF in 2010, Lower Limit | PAF in 2010, Upper Limit | PAF in 2014 | PAF in 2014, Lower Limit | PAF in 2014, Upper Limit |
| --- | --- | --- | --- | --- | --- | --- | --- | --- | --- | --- | --- | --- | --- | --- | --- | --- | --- | --- | --- |
| Bolivia | Men | 0.2175 | 0.2102 | 0.2253 | 0.2824 | 0.2751 | 0.2891 | 0.3492 | 0.3415 | 0.3572 | 0.3805 | 0.3717 | 0.3881 | 0.4125 | 0.4037 | 0.4206 | 0.4386 | 0.4295 | 0.4468 |
| Bolivia | Women | 0.3457 | 0.3368 | 0.3539 | 0.4216 | 0.4125 | 0.4310 | 0.4907 | 0.4812 | 0.4996 | 0.5207 | 0.5110 | 0.5299 | 0.5495 | 0.5403 | 0.5589 | 0.5717 | 0.5618 | 0.5814 |
| Ecuador | Men | 0.2323 | 0.2249 | 0.2397 | 0.2965 | 0.2895 | 0.3046 | 0.3603 | 0.3523 | 0.3682 | 0.3905 | 0.3828 | 0.3989 | 0.4211 | 0.4121 | 0.4300 | 0.4458 | 0.4373 | 0.4556 |
| Ecuador | Women | 0.3501 | 0.3422 | 0.3591 | 0.4211 | 0.4121 | 0.4303 | 0.4834 | 0.4737 | 0.4924 | 0.5119 | 0.5028 | 0.5213 | 0.5377 | 0.5284 | 0.5469 | 0.5587 | 0.5490 | 0.5683 |
| Peru | Men | 0.2615 | 0.2543 | 0.2689 | 0.3162 | 0.3091 | 0.3235 | 0.3720 | 0.3639 | 0.3790 | 0.3996 | 0.3918 | 0.4076 | 0.4275 | 0.4192 | 0.4352 | 0.4500 | 0.4416 | 0.4585 |
| Peru | Women | 0.3859 | 0.3771 | 0.3945 | 0.4364 | 0.4282 | 0.4447 | 0.4844 | 0.4752 | 0.4930 | 0.5084 | 0.4994 | 0.5175 | 0.5322 | 0.5232 | 0.5421 | 0.5516 | 0.5411 | 0.5609 |

| Country | Sex | PAF in 2014 (25-29 BMI) | PAF in 2014 (25-29 BMI), Lower Limit | PAF in 2014 (25-29 BMI), Upper Limit | PAF in 2014 (30-34 BMI) | PAF in 2014 (30-34 BMI), Lower Limit | PAF in 2014 (30-34 BMI), Upper Limit | PAF in 2014 (35-39 BMI) | PAF in 2014 (35-39 BMI), Lower Limit | PAF in 2014 (35-39 BMI), Upper Limit | PAF in 2014 (40+ BMI) | PAF in 2014 (40+ BMI), Lower Limit | PAF in 2014 (40+ BMI), Upper Limit |
| --- | --- | --- | --- | --- | --- | --- | --- | --- | --- | --- | --- | --- | --- |
| Bolivia | Men | 0.2518 | 0.2466 | 0.2568 | 0.2351 | 0.2280 | 0.2418 | 0.0800 | 0.0767 | 0.0834 | 0.0482 | 0.0457 | 0.0506 |
| Bolivia | Women | 0.2341 | 0.2292 | 0.2390 | 0.3036 | 0.2959 | 0.3114 | 0.2549 | 0.2463 | 0.2641 | 0.2007 | 0.1919 | 0.2099 |
| Ecuador | Men | 0.2522 | 0.2472 | 0.2578 | 0.2364 | 0.2300 | 0.2437 | 0.0925 | 0.0889 | 0.0965 | 0.0527 | 0.0501 | 0.0556 |
| Ecuador | Women | 0.2356 | 0.2307 | 0.2404 | 0.3047 | 0.2971 | 0.3125 | 0.2382 | 0.2299 | 0.2465 | 0.1715 | 0.1636 | 0.1794 |
| Peru | Men | 0.2612 | 0.2560 | 0.2663 | 0.2438 | 0.2375 | 0.2507 | 0.0918 | 0.0882 | 0.0956 | 0.0394 | 0.0374 | 0.0415 |
| Peru | Women | 0.2428 | 0.2374 | 0.2478 | 0.3069 | 0.2988 | 0.3146 | 0.2309 | 0.2224 | 0.2389 | 0.1426 | 0.1357 | 0.1493 |

| Country | Sex | PAF in 2010 (25-29 BMI) | PAF in 2010 (25-29 BMI), Lower Limit | PAF in 2010 (25-29 BMI), Upper Limit | PAF in 2010 (30-34 BMI) | PAF in 2010 (30-34 BMI), Lower Limit | PAF in 2010 (30-34 BMI), Upper Limit | PAF in 2010 (35-39 BMI) | PAF in 2010 (35-39 BMI), Lower Limit | PAF in 2010 (35-39 BMI), Upper Limit | PAF in 2010 (40+ BMI) | PAF in 2010 (40+ BMI), Lower Limit | PAF in 2010 (40+ BMI), Upper Limit |
| --- | --- | --- | --- | --- | --- | --- | --- | --- | --- | --- | --- | --- | --- |
| Bolivia | Men | 0.2461 | 0.2407 | 0.2512 | 0.2160 | 0.2095 | 0.2223 | 0.0624 | 0.0597 | 0.0651 | 0.0326 | 0.0308 | 0.0343 |
| Bolivia | Women | 0.2336 | 0.2289 | 0.2386 | 0.2928 | 0.2858 | 0.3004 | 0.2297 | 0.2217 | 0.2378 | 0.1682 | 0.1607 | 0.1758 |
| Ecuador | Men | 0.2476 | 0.2424 | 0.2529 | 0.2195 | 0.2128 | 0.2262 | 0.0737 | 0.0706 | 0.0769 | 0.0358 | 0.0338 | 0.0377 |
| Ecuador | Women | 0.2349 | 0.2299 | 0.2397 | 0.2943 | 0.2867 | 0.3015 | 0.2146 | 0.2073 | 0.2224 | 0.1424 | 0.1357 | 0.1493 |
| Peru | Men | 0.2561 | 0.2508 | 0.2610 | 0.2268 | 0.2206 | 0.2328 | 0.0742 | 0.0711 | 0.0772 | 0.0281 | 0.0266 | 0.0296 |
| Peru | Women | 0.2421 | 0.2373 | 0.2474 | 0.2966 | 0.2896 | 0.3044 | 0.2077 | 0.2008 | 0.2156 | 0.1185 | 0.1132 | 0.1247 |

| Country | Sex | PAF in 2005 (25-29 BMI) | PAF in 2005 (25-29 BMI), Lower Limit | PAF in 2005 (25-29 BMI), Upper Limit | PAF in 2005 (30-34 BMI) | PAF in 2005 (30-34 BMI), Lower Limit | PAF in 2005 (30-34 BMI), Upper Limit | PAF in 2005 (35-39 BMI) | PAF in 2005 (35-39 BMI), Lower Limit | PAF in 2005 (35-39 BMI), Upper Limit | PAF in 2005 (40+ BMI) | PAF in 2005 (40+ BMI), Lower Limit | PAF in 2005 (40+ BMI), Upper Limit |
| --- | --- | --- | --- | --- | --- | --- | --- | --- | --- | --- | --- | --- | --- |
| Bolivia | Men | 0.2370 | 0.2320 | 0.2418 | 0.1912 | 0.1850 | 0.1970 | 0.0447 | 0.0428 | 0.0467 | 0.0196 | 0.0185 | 0.0206 |
| Bolivia | Women | 0.2319 | 0.2269 | 0.2365 | 0.2779 | 0.2702 | 0.2849 | 0.1988 | 0.1912 | 0.2058 | 0.1316 | 0.1254 | 0.1378 |
| Ecuador | Men | 0.2399 | 0.2351 | 0.2449 | 0.1972 | 0.1914 | 0.2032 | 0.0545 | 0.0523 | 0.0569 | 0.0217 | 0.0206 | 0.0229 |
| Ecuador | Women | 0.2332 | 0.2286 | 0.2380 | 0.2802 | 0.2732 | 0.2876 | 0.1867 | 0.1801 | 0.1933 | 0.1116 | 0.1067 | 0.1171 |
| Peru | Men | 0.2482 | 0.2432 | 0.2536 | 0.2047 | 0.1991 | 0.2108 | 0.0559 | 0.0537 | 0.0585 | 0.0181 | 0.0172 | 0.0191 |
| Peru | Women | 0.2405 | 0.2356 | 0.2454 | 0.2829 | 0.2760 | 0.2903 | 0.1804 | 0.1739 | 0.1872 | 0.0933 | 0.0889 | 0.0981 |

| Country | Sex | PAF in 2000 (25-29 BMI) | PAF in 2000 (25-29 BMI), Lower Limit | PAF in 2000 (25-29 BMI), Upper Limit | PAF in 2000 (30-34 BMI) | PAF in 2000 (30-34 BMI), Lower Limit | PAF in 2000 (30-34 BMI), Upper Limit | PAF in 2000 (35-39 BMI) | PAF in 2000 (35-39 BMI), Lower Limit | PAF in 2000 (35-39 BMI), Upper Limit | PAF in 2000 (40+ BMI) | PAF in 2000 (40+ BMI), Lower Limit | PAF in 2000 (40+ BMI), Upper Limit |
| --- | --- | --- | --- | --- | --- | --- | --- | --- | --- | --- | --- | --- | --- |
| Bolivia | Men | 0.2266 | 0.2219 | 0.2319 | 0.1662 | 0.1611 | 0.1716 | 0.0313 | 0.0300 | 0.0328 | 0.0115 | 0.0109 | 0.0121 |
| Bolivia | Women | 0.2289 | 0.2241 | 0.2335 | 0.2607 | 0.2538 | 0.2675 | 0.1685 | 0.1621 | 0.1748 | 0.1001 | 0.0952 | 0.1048 |
| Ecuador | Men | 0.2306 | 0.2255 | 0.2355 | 0.1736 | 0.1683 | 0.1789 | 0.0390 | 0.0373 | 0.0407 | 0.0128 | 0.0121 | 0.0135 |
| Ecuador | Women | 0.2299 | 0.2251 | 0.2346 | 0.2628 | 0.2556 | 0.2699 | 0.1584 | 0.1522 | 0.1644 | 0.0846 | 0.0805 | 0.0888 |
| Peru | Men | 0.2393 | 0.2341 | 0.2441 | 0.1824 | 0.1769 | 0.1874 | 0.0413 | 0.0395 | 0.0430 | 0.0113 | 0.0107 | 0.0119 |
| Peru | Women | 0.2379 | 0.2330 | 0.2426 | 0.2678 | 0.2610 | 0.2745 | 0.1549 | 0.1491 | 0.1606 | 0.0726 | 0.0689 | 0.0762 |

| Country | Sex | PAF in 1990 (25-29 BMI) | PAF in 1990 (25-29 BMI), Lower Limit | PAF in 1990 (25-29 BMI), Upper Limit | PAF in 1990 (30-34 BMI) | PAF in 1990 (30-34 BMI), Lower Limit | PAF in 1990 (30-34 BMI), Upper Limit | PAF in 1990 (35-39 BMI) | PAF in 1990 (35-39 BMI), Lower Limit | PAF in 1990 (35-39 BMI), Upper Limit | PAF in 1990 (40+ BMI) | PAF in 1990 (40+ BMI), Lower Limit | PAF in 1990 (40+ BMI), Upper Limit |
| --- | --- | --- | --- | --- | --- | --- | --- | --- | --- | --- | --- | --- | --- |
| Bolivia | Men | 0.1998 | 0.1951 | 0.2045 | 0.1129 | 0.1093 | 0.1166 | 0.0129 | 0.0123 | 0.0135 | 0.0033 | 0.0031 | 0.0034 |
| Bolivia | Women | 0.2174 | 0.2125 | 0.2222 | 0.2171 | 0.2106 | 0.2237 | 0.1089 | 0.1043 | 0.1134 | 0.0491 | 0.0464 | 0.0518 |
| Ecuador | Men | 0.2066 | 0.2018 | 0.2117 | 0.1226 | 0.1187 | 0.1267 | 0.0173 | 0.0165 | 0.0181 | 0.0040 | 0.0037 | 0.0042 |
| Ecuador | Women | 0.2188 | 0.2139 | 0.2240 | 0.2222 | 0.2156 | 0.2289 | 0.1048 | 0.1004 | 0.1091 | 0.0428 | 0.0406 | 0.0453 |
| Peru | Men | 0.2181 | 0.2132 | 0.2228 | 0.1372 | 0.1332 | 0.1417 | 0.0203 | 0.0194 | 0.0212 | 0.0038 | 0.0036 | 0.0040 |
| Peru | Women | 0.2300 | 0.2252 | 0.2344 | 0.2355 | 0.2293 | 0.2418 | 0.1108 | 0.1066 | 0.1152 | 0.0414 | 0.0394 | 0.0436 |

| Country | Sex | PAF in 1980 (25-29 BMI) | PAF in 1980 (25-29 BMI), Lower Limit | PAF in 1980 (25-29 BMI), Upper Limit | PAF in 1980 (30-34 BMI) | PAF in 1980 (30-34 BMI), Lower Limit | PAF in 1980 (30-34 BMI), Upper Limit | PAF in 1980 (35-39 BMI) | PAF in 1980 (35-39 BMI), Lower Limit | PAF in 1980 (35-39 BMI), Upper Limit | PAF in 1980 (40+ BMI) | PAF in 1980 (40+ BMI), Lower Limit | PAF in 1980 (40+ BMI), Upper Limit | Number people with diabetes in 2014 |
| --- | --- | --- | --- | --- | --- | --- | --- | --- | --- | --- | --- | --- | --- | --- |
| Bolivia | Men | 0.1676 | 0.1634 | 0.1722 | 0.0664 | 0.0642 | 0.0688 | 0.0045 | 0.0043 | 0.0047 | 0.0009 | 0.0008 | 0.0009 | 175226 |
| Bolivia | Women | 0.1973 | 0.1924 | 0.2017 | 0.1653 | 0.1596 | 0.1708 | 0.0605 | 0.0580 | 0.0631 | 0.0197 | 0.0187 | 0.0208 | 250369 |
| Ecuador | Men | 0.1762 | 0.1718 | 0.1807 | 0.0751 | 0.0725 | 0.0776 | 0.0064 | 0.0061 | 0.0067 | 0.0011 | 0.0011 | 0.0012 | 344013 |
| Ecuador | Women | 0.1994 | 0.1951 | 0.2046 | 0.1724 | 0.1670 | 0.1781 | 0.0596 | 0.0573 | 0.0624 | 0.0177 | 0.0168 | 0.0187 | 413954 |
| Peru | Men | 0.1929 | 0.1884 | 0.1975 | 0.0949 | 0.0919 | 0.0984 | 0.0089 | 0.0085 | 0.0093 | 0.0012 | 0.0011 | 0.0013 | 644349 |
| Peru | Women | 0.2171 | 0.2120 | 0.2221 | 0.1992 | 0.1929 | 0.2054 | 0.0745 | 0.0713 | 0.0777 | 0.0216 | 0.0204 | 0.0229 | 762085 |

# **Population attributable fraction cases by regions in Peru**

The number of attributable cases of diabetes in 2017 were computed using body mass index (BMI) distribution in 2014. The PAF were computed for a 5-unit change in BMI and for different BMI ranges.

| Region | PAF 2014 | PAF 2014, Lower Limit | PAF 2014, Upper Limit | PAF 2014 (25-29 BMI) | PAF 2014 (25-29 BMI), Lower Limit | PAF 2014 (25-29 BMI), Upper Limit | PAF 2014 (30-34 BMI) | PAF 2014 (30-34 BMI), Lower Limit | PAF 2014 (30-34 BMI), Upper Limit | PAF 2014 (35-40 BMI) | PAF 2014 (35-40 BMI), Lower Limit | PAF 2014 (35-40 BMI), Upper Limit | PAF 2014 (40+ BMI) | PAF 2014 (40+ BMI), Lower Limit | PAF 2014 (40+ BMI), Upper Limit | Prevalence of Diabetes in 2017 | People with diabetes in 2017 | Population |
| --- | --- | --- | --- | --- | --- | --- | --- | --- | --- | --- | --- | --- | --- | --- | --- | --- | --- | --- |
| Amazonas | 0.0795 | 0.0670 | 0.0922 | 0.1656 | 0.1617 | 0.1695 | 0.1571 | 0.1520 | 0.1622 | 0.0459 | 0.0439 | 0.0479 | 0.0000 | 0.0000 | 0.0000 | 0.0652 | 14753 | 226193 |
| Ancash | 0.2636 | 0.2505 | 0.2782 | 0.1752 | 0.1717 | 0.1792 | 0.1961 | 0.1910 | 0.2019 | 0.1267 | 0.1221 | 0.1318 | 0.0937 | 0.0894 | 0.0986 | 0.0746 | 52528 | 704284 |
| Apurimac | 0.0000 | 0.0000 | 0.0047 | 0.1626 | 0.1586 | 0.1664 | 0.1072 | 0.1035 | 0.1107 | 0.0253 | 0.0242 | 0.0265 | 0.0000 | 0.0000 | 0.0000 | 0.0409 | 10412 | 254364 |
| Arequipa | 0.3232 | 0.3090 | 0.3365 | 0.2138 | 0.2092 | 0.2182 | 0.2069 | 0.2010 | 0.2128 | 0.1858 | 0.1788 | 0.1926 | 0.0592 | 0.0560 | 0.0624 | 0.0572 | 53724 | 939624 |
| Ayacucho | 0.0658 | 0.0535 | 0.0788 | 0.1669 | 0.1631 | 0.1710 | 0.1363 | 0.1322 | 0.1408 | 0.0396 | 0.0379 | 0.0413 | 0.0173 | 0.0164 | 0.0183 | 0.0643 | 24599 | 382437 |
| Cajamarca | 0.0281 | 0.0163 | 0.0408 | 0.1738 | 0.1701 | 0.1777 | 0.0883 | 0.0855 | 0.0912 | 0.0401 | 0.0384 | 0.0419 | 0.0258 | 0.0245 | 0.0273 | 0.0494 | 41514 | 840133 |
| Callao | 0.3771 | 0.3623 | 0.3924 | 0.1926 | 0.1880 | 0.1972 | 0.2875 | 0.2801 | 0.2952 | 0.1576 | 0.1514 | 0.1644 | 0.1093 | 0.1038 | 0.1154 | 0.0674 | 44898 | 666085 |
| Cusco | 0.1324 | 0.1188 | 0.1451 | 0.1757 | 0.1717 | 0.1796 | 0.1544 | 0.1497 | 0.1588 | 0.0653 | 0.0624 | 0.0683 | 0.0324 | 0.0302 | 0.0346 | 0.0532 | 40791 | 766773 |
| Huancavelica | 0.0269 | 0.0141 | 0.0403 | 0.1513 | 0.1478 | 0.1548 | 0.1065 | 0.1032 | 0.1099 | 0.0621 | 0.0584 | 0.0666 | 0.0105 | 0.0099 | 0.0111 | 0.0343 | 7253 | 211664 |
| Huanuco | 0.0152 | 0.0022 | 0.0271 | 0.1518 | 0.1481 | 0.1553 | 0.0848 | 0.0819 | 0.0875 | 0.0621 | 0.0592 | 0.0649 | 0.0236 | 0.0224 | 0.0249 | 0.0387 | 17003 | 439724 |
| Ica | 0.3595 | 0.3459 | 0.3732 | 0.2012 | 0.1968 | 0.2057 | 0.2864 | 0.2790 | 0.2938 | 0.1420 | 0.1365 | 0.1479 | 0.0819 | 0.0779 | 0.0863 | 0.0643 | 34896 | 542940 |
| Junin | 0.1569 | 0.1432 | 0.1719 | 0.1887 | 0.1845 | 0.1933 | 0.1206 | 0.1167 | 0.1247 | 0.0990 | 0.0949 | 0.1035 | 0.0506 | 0.0480 | 0.0534 | 0.0370 | 29003 | 783716 |
| La Libertad | 0.3029 | 0.2884 | 0.3179 | 0.1849 | 0.1807 | 0.1892 | 0.2059 | 0.2003 | 0.2117 | 0.1692 | 0.1630 | 0.1757 | 0.0837 | 0.0795 | 0.0879 | 0.0425 | 47978 | 1130064 |
| Lambayeque | 0.3419 | 0.3275 | 0.3559 | 0.1977 | 0.1933 | 0.2021 | 0.2196 | 0.2136 | 0.2256 | 0.1809 | 0.1742 | 0.1877 | 0.1052 | 0.1000 | 0.1105 | 0.0651 | 49748 | 763863 |
| Lima | 0.3828 | 0.3691 | 0.3968 | 0.2046 | 0.2003 | 0.2091 | 0.2653 | 0.2586 | 0.2722 | 0.1879 | 0.1812 | 0.1949 | 0.1049 | 0.0998 | 0.1102 | 0.0584 | 385469 | 6596560 |
| Loreto | 0.1531 | 0.1383 | 0.1670 | 0.1788 | 0.1744 | 0.1827 | 0.1432 | 0.1384 | 0.1477 | 0.0790 | 0.0753 | 0.0823 | 0.0539 | 0.0509 | 0.0569 | 0.0728 | 35521 | 488087 |
| Mdr Dios | 0.3221 | 0.3081 | 0.3353 | 0.2114 | 0.2067 | 0.2157 | 0.2480 | 0.2412 | 0.2540 | 0.1251 | 0.1201 | 0.1299 | 0.0752 | 0.0712 | 0.0789 | 0.0853 | 7320 | 85794 |
| Moquegua | 0.3818 | 0.3674 | 0.3958 | 0.2079 | 0.2032 | 0.2122 | 0.2604 | 0.2535 | 0.2671 | 0.1539 | 0.1480 | 0.1595 | 0.1436 | 0.1370 | 0.1503 | 0.0828 | 10008 | 120929 |
| Pasco | 0.0726 | 0.0604 | 0.0857 | 0.1715 | 0.1678 | 0.1755 | 0.1222 | 0.1184 | 0.1259 | 0.0478 | 0.0459 | 0.0499 | 0.0278 | 0.0264 | 0.0294 | 0.0336 | 5475 | 163121 |
| Piura | 0.2958 | 0.2811 | 0.3099 | 0.1871 | 0.1829 | 0.1912 | 0.2182 | 0.2120 | 0.2242 | 0.1185 | 0.1140 | 0.1234 | 0.1095 | 0.1041 | 0.1149 | 0.0749 | 85915 | 1147498 |
| Puno | 0.1289 | 0.1156 | 0.1427 | 0.1603 | 0.1566 | 0.1642 | 0.1398 | 0.1356 | 0.1444 | 0.1210 | 0.1162 | 0.1262 | 0.0034 | 0.0032 | 0.0036 | 0.0567 | 44207 | 779583 |
| San Martin | 0.1129 | 0.0996 | 0.1274 | 0.1811 | 0.1770 | 0.1855 | 0.1643 | 0.1590 | 0.1697 | 0.0054 | 0.0052 | 0.0057 | 0.0485 | 0.0460 | 0.0514 | 0.0537 | 26357 | 490997 |
| Tacna | 0.3762 | 0.3627 | 0.3900 | 0.1841 | 0.1799 | 0.1883 | 0.3087 | 0.3013 | 0.3162 | 0.1395 | 0.1342 | 0.1448 | 0.1034 | 0.0984 | 0.1086 | 0.0844 | 19004 | 225066 |
| Tumbes | 0.2982 | 0.2832 | 0.3125 | 0.1868 | 0.1825 | 0.1912 | 0.2325 | 0.2256 | 0.2389 | 0.1323 | 0.1270 | 0.1376 | 0.0795 | 0.0754 | 0.0839 | 0.0795 | 11193 | 140820 |
| Ucayali | 0.2792 | 0.2665 | 0.2929 | 0.1852 | 0.1813 | 0.1894 | 0.2283 | 0.2226 | 0.2346 | 0.1560 | 0.1504 | 0.1622 | 0.0251 | 0.0236 | 0.0267 | 0.0446 | 12688 | 284491 |

# **Population attributable fraction and number of attributable cases by region in Peru in men**

The number of attributable cases of diabetes in 2017 were computed using body mass index (BMI) distribution in 2014.

| Region | PAF 2014, (men) | PAF 2014, Lower Limit (men) | PAF 2014, Upper Limit (men) | PAF 2014 (25-29 BMI), (men) | PAF 2014 (25-29 BMI), Lower Limit (men) | PAF 2014 (25-29 BMI), Upper Limit (men) | PAF 2014 (30-34 BMI), (men) | PAF 2014 (30-34 BMI), Lower Limit (men) | PAF 2014 (30-34 BMI), Upper Limit (men) | PAF 2014 (35-39 BMI), (men) | PAF 2014 (35-39 BMI), Lower Limit (men) | PAF 2014 (35-39 BMI), Upper Limit (men) | PAF 2014 (40+ BMI), (men) | PAF 2014 (40+ BMI), Lower Limit (men) | PAF 2014 (40+ BMI), Upper Limit (men) | Prevalence of Diabetes (men) | Number people with diabetes 2017 (men) | Number people with diabetes 2017, PAF 2014, (men) | Number people with diabetes 2017, PAF 2014 (25-29 BMI), (men) | Number people with diabetes 2017, PAF 2014 (30-34 BMI), (men) | Number people with diabetes 2017, PAF 2014 (35-39 BMI), (men) | Number people with diabetes 2017, PAF 2014 (40+ BMI), (men) |
| --- | --- | --- | --- | --- | --- | --- | --- | --- | --- | --- | --- | --- | --- | --- | --- | --- | --- | --- | --- | --- | --- | --- |
| Amazonas | 0.0000 | 0.0000 | 0.0000 | 0.1544 | 0.1507 | 0.1580 | 0.1080 | 0.1040 | 0.1121 | 0.0117 | 0.0111 | 0.0124 | 0.0000 | 0.0000 | 0.0000 | 0.0692 | 7872 | 0 | 1215 | 849 | 92 | 0 |
| Ancash | 0.1529 | 0.1407 | 0.1666 | 0.1760 | 0.1723 | 0.1800 | 0.1603 | 0.1558 | 0.1655 | 0.1175 | 0.1132 | 0.1225 | 0.0000 | 0.0000 | 0.0000 | 0.0665 | 22671 | 3465 | 3989 | 3633 | 2663 | 0 |
| Apurimac | 0.0000 | 0.0000 | 0.0000 | 0.1489 | 0.1452 | 0.1525 | 0.1031 | 0.0995 | 0.1065 | 0.0071 | 0.0067 | 0.0075 | 0.0000 | 0.0000 | 0.0000 | 0.0539 | 6651 | 0 | 990 | 685 | 47 | 0 |
| Arequipa | 0.2228 | 0.2089 | 0.2364 | 0.2160 | 0.2111 | 0.2206 | 0.1825 | 0.1769 | 0.1881 | 0.0869 | 0.0831 | 0.0906 | 0.0475 | 0.0446 | 0.0505 | 0.0705 | 31994 | 7128 | 6911 | 5839 | 2778 | 1519 |
| Ayacucho | 0.0000 | 0.0000 | 0.0057 | 0.1587 | 0.1550 | 0.1628 | 0.1221 | 0.1181 | 0.1264 | 0.0221 | 0.0211 | 0.0231 | 0.0000 | 0.0000 | 0.0000 | 0.0462 | 8553 | 0 | 1357 | 1043 | 188 | 0 |
| Cajamarca | 0.0000 | 0.0000 | 0.0000 | 0.1700 | 0.1663 | 0.1739 | 0.0732 | 0.0707 | 0.0758 | 0.0194 | 0.0184 | 0.0204 | 0.0078 | 0.0074 | 0.0082 | 0.0469 | 18921 | 0 | 3215 | 1385 | 367 | 146 |
| Callao | 0.3017 | 0.2871 | 0.3176 | 0.1935 | 0.1890 | 0.1983 | 0.2635 | 0.2565 | 0.2709 | 0.1075 | 0.1028 | 0.1125 | 0.0665 | 0.0630 | 0.0707 | 0.0649 | 20749 | 6260 | 4015 | 5466 | 2230 | 1379 |
| Cusco | 0.0768 | 0.0627 | 0.0904 | 0.1796 | 0.1754 | 0.1836 | 0.1207 | 0.1165 | 0.1244 | 0.0339 | 0.0316 | 0.0360 | 0.0467 | 0.0429 | 0.0504 | 0.0509 | 19074 | 1464 | 3424 | 2302 | 645 | 890 |
| Huancavelica | 0.0000 | 0.0000 | 0.0000 | 0.1559 | 0.1523 | 0.1595 | 0.0770 | 0.0736 | 0.0802 | 0.0108 | 0.0103 | 0.0114 | 0.0000 | 0.0000 | 0.0000 | 0.0273 | 2723 | 0 | 424 | 209 | 29 | 0 |
| Huanuco | 0.0000 | 0.0000 | 0.0000 | 0.1505 | 0.1467 | 0.1540 | 0.0501 | 0.0482 | 0.0517 | 0.0454 | 0.0427 | 0.0481 | 0.0000 | 0.0000 | 0.0000 | 0.0237 | 5067 | 0 | 762 | 253 | 229 | 0 |
| Ica | 0.3061 | 0.2921 | 0.3197 | 0.1978 | 0.1933 | 0.2023 | 0.2832 | 0.2758 | 0.2908 | 0.0814 | 0.0780 | 0.0851 | 0.0636 | 0.0603 | 0.0670 | 0.0607 | 15970 | 4888 | 3158 | 4522 | 1300 | 1015 |
| Junin | 0.0402 | 0.0275 | 0.0544 | 0.1903 | 0.1861 | 0.1951 | 0.0786 | 0.0759 | 0.0815 | 0.0609 | 0.0583 | 0.0638 | 0.0137 | 0.0129 | 0.0146 | 0.0225 | 8403 | 337 | 1599 | 660 | 511 | 115 |
| La Libertad | 0.1938 | 0.1795 | 0.2082 | 0.1898 | 0.1856 | 0.1942 | 0.1750 | 0.1695 | 0.1808 | 0.1097 | 0.1053 | 0.1143 | 0.0271 | 0.0257 | 0.0286 | 0.0380 | 20525 | 3977 | 3896 | 3591 | 2251 | 556 |
| Lambayeque | 0.2171 | 0.2030 | 0.2307 | 0.2024 | 0.1979 | 0.2069 | 0.2071 | 0.2012 | 0.2131 | 0.0592 | 0.0564 | 0.0622 | 0.0533 | 0.0504 | 0.0562 | 0.0648 | 23313 | 5062 | 4718 | 4828 | 1379 | 1241 |
| Lima | 0.3126 | 0.2990 | 0.3265 | 0.2115 | 0.2071 | 0.2160 | 0.2437 | 0.2374 | 0.2501 | 0.1270 | 0.1222 | 0.1321 | 0.0707 | 0.0671 | 0.0743 | 0.0484 | 153333 | 47935 | 32433 | 37360 | 19478 | 10833 |
| Loreto | 0.1286 | 0.1130 | 0.1423 | 0.1849 | 0.1804 | 0.1888 | 0.1219 | 0.1177 | 0.1259 | 0.0870 | 0.0828 | 0.0908 | 0.0418 | 0.0393 | 0.0443 | 0.0532 | 12927 | 1662 | 2390 | 1575 | 1124 | 539 |
| Mdr Dios | 0.2578 | 0.2439 | 0.2714 | 0.2163 | 0.2115 | 0.2206 | 0.2339 | 0.2273 | 0.2399 | 0.0443 | 0.0424 | 0.0461 | 0.0717 | 0.0678 | 0.0755 | 0.0735 | 3352 | 864 | 724 | 783 | 148 | 240 |
| Moquegua | 0.2805 | 0.2663 | 0.2940 | 0.2125 | 0.2078 | 0.2169 | 0.2381 | 0.2316 | 0.2444 | 0.0795 | 0.0762 | 0.0828 | 0.0723 | 0.0679 | 0.0766 | 0.0856 | 5185 | 1454 | 1101 | 1234 | 412 | 375 |
| Pasco | 0.0130 | 0.0006 | 0.0264 | 0.1599 | 0.1564 | 0.1638 | 0.0986 | 0.0952 | 0.1020 | 0.0359 | 0.0343 | 0.0376 | 0.0322 | 0.0307 | 0.0340 | 0.0158 | 1288 | 16 | 205 | 127 | 46 | 41 |
| Piura | 0.2074 | 0.1930 | 0.2211 | 0.1939 | 0.1895 | 0.1983 | 0.1917 | 0.1861 | 0.1972 | 0.0657 | 0.0630 | 0.0685 | 0.0661 | 0.0625 | 0.0696 | 0.0751 | 42018 | 8715 | 8149 | 8053 | 2758 | 2777 |
| Puno | 0.0140 | 0.0015 | 0.0263 | 0.1758 | 0.1716 | 0.1802 | 0.1185 | 0.1148 | 0.1224 | 0.0229 | 0.0218 | 0.0240 | 0.0000 | 0.0000 | 0.0000 | 0.0410 | 15526 | 216 | 2729 | 1839 | 355 | 0 |
| San Martin | 0.0662 | 0.0533 | 0.0806 | 0.1800 | 0.1759 | 0.1844 | 0.1423 | 0.1376 | 0.1473 | 0.0068 | 0.0065 | 0.0071 | 0.0345 | 0.0326 | 0.0365 | 0.0459 | 11514 | 762 | 2072 | 1638 | 78 | 396 |
| Tacna | 0.2932 | 0.2794 | 0.3069 | 0.1942 | 0.1897 | 0.1985 | 0.2693 | 0.2624 | 0.2762 | 0.0892 | 0.0856 | 0.0928 | 0.0604 | 0.0573 | 0.0636 | 0.0741 | 8246 | 2417 | 1601 | 2220 | 735 | 497 |
| Tumbes | 0.2387 | 0.2244 | 0.2525 | 0.1832 | 0.1789 | 0.1876 | 0.2459 | 0.2386 | 0.2524 | 0.0694 | 0.0663 | 0.0725 | 0.0438 | 0.0405 | 0.0471 | 0.0643 | 4548 | 1085 | 833 | 1118 | 315 | 199 |
| Ucayali | 0.2522 | 0.2395 | 0.2666 | 0.1933 | 0.1891 | 0.1976 | 0.2226 | 0.2169 | 0.2291 | 0.1189 | 0.1145 | 0.1239 | 0.0324 | 0.0304 | 0.0348 | 0.0375 | 5375 | 1355 | 1038 | 1196 | 639 | 174 |

# **Population attributable fraction and number of attributable cases by region in Peru in women**

The number of attributable cases of diabetes in 2017 were computed using body mass index (BMI) distribution in 2014.

| Region | PAF 2014, (women) | PAF 2014, Lower Limit (women) | PAF 2014, Upper Limit (women) | PAF 2014 (25-29 BMI), (women) | PAF 2014 (25-29 BMI), Lower Limit (women) | PAF 2014 (25-29 BMI), Upper Limit (women) | PAF 2014 (30-34 BMI), (women) | PAF 2014 (30-34 BMI), Lower Limit (women) | PAF 2014 (30-34 BMI), Upper Limit (women) | PAF 2014 (35-39 BMI), (women) | PAF 2014 (35-39 BMI), Lower Limit (women) | PAF 2014 (35-39 BMI), Upper Limit (women) | PAF 2014 (40+ BMI), (women) | PAF 2014 (40+ BMI), Lower Limit (women) | PAF 2014 (40+ BMI), Upper Limit (women) | Prevalence of Diabetes (women) | Number people with diabetes 2017 (women) | Number people with diabetes 2017, PAF 2014, (women) | Number people with diabetes 2017, PAF 2014 (25-29 BMI), (women) | Number people with diabetes 2017, PAF 2014 (30-34 BMI), (women) | Number people with diabetes 2017, PAF 2014 (35-39 BMI), (women) | Number people with diabetes 2017, PAF 2014 (40+ BMI), (women) |
| --- | --- | --- | --- | --- | --- | --- | --- | --- | --- | --- | --- | --- | --- | --- | --- | --- | --- | --- | --- | --- | --- | --- |
| Amazonas | 0.2990 | 0.2797 | 0.3176 | 0.1929 | 0.1873 | 0.1987 | 0.2710 | 0.2594 | 0.2825 | 0.1323 | 0.1266 | 0.1385 | 0.0000 | 0.0000 | 0.0000 | 0.0609 | 6843 | 2046 | 1319 | 1854 | 905 | 0 |
| Ancash | 0.4263 | 0.4120 | 0.4427 | 0.1703 | 0.1663 | 0.1747 | 0.2662 | 0.2580 | 0.2757 | 0.1451 | 0.1390 | 0.1520 | 0.2551 | 0.2443 | 0.2670 | 0.0823 | 29910 | 12751 | 5092 | 7960 | 4338 | 7629 |
| Apurimac | 0.0918 | 0.0775 | 0.1062 | 0.1912 | 0.1865 | 0.1962 | 0.1148 | 0.1106 | 0.1190 | 0.0668 | 0.0637 | 0.0701 | 0.0000 | 0.0000 | 0.0000 | 0.0286 | 3741 | 343 | 715 | 429 | 249 | 0 |
| Arequipa | 0.4755 | 0.4609 | 0.4904 | 0.2043 | 0.1991 | 0.2095 | 0.2575 | 0.2499 | 0.2650 | 0.3535 | 0.3413 | 0.3665 | 0.0852 | 0.0806 | 0.0899 | 0.0440 | 21395 | 10173 | 4371 | 5508 | 7562 | 1822 |
| Ayacucho | 0.1901 | 0.1756 | 0.2056 | 0.1832 | 0.1788 | 0.1880 | 0.1668 | 0.1611 | 0.1732 | 0.0795 | 0.0762 | 0.0833 | 0.0570 | 0.0537 | 0.0607 | 0.0809 | 15967 | 3035 | 2924 | 2663 | 1269 | 910 |
| Cajamarca | 0.1728 | 0.1542 | 0.1897 | 0.1803 | 0.1747 | 0.1861 | 0.1261 | 0.1208 | 0.1313 | 0.0930 | 0.0885 | 0.0978 | 0.0727 | 0.0687 | 0.0768 | 0.0518 | 22624 | 3909 | 4080 | 2852 | 2103 | 1644 |
| Callao | 0.5001 | 0.4852 | 0.5155 | 0.1862 | 0.1815 | 0.1909 | 0.3372 | 0.3283 | 0.3468 | 0.2591 | 0.2490 | 0.2696 | 0.1996 | 0.1898 | 0.2102 | 0.0698 | 24198 | 12102 | 4505 | 8158 | 6269 | 4828 |
| Cusco | 0.2170 | 0.2011 | 0.2330 | 0.1647 | 0.1606 | 0.1689 | 0.2156 | 0.2079 | 0.2225 | 0.1243 | 0.1152 | 0.1334 | 0.0000 | 0.0000 | 0.0000 | 0.0554 | 21727 | 4714 | 3578 | 4684 | 2701 | 0 |
| Huancavelica | 0.2046 | 0.1768 | 0.2310 | 0.1370 | 0.1330 | 0.1411 | 0.1680 | 0.1624 | 0.1735 | 0.1647 | 0.1424 | 0.1862 | 0.0343 | 0.0321 | 0.0368 | 0.0407 | 4552 | 931 | 623 | 764 | 749 | 156 |
| Huanuco | 0.2044 | 0.1864 | 0.2220 | 0.1518 | 0.1467 | 0.1564 | 0.1696 | 0.1624 | 0.1757 | 0.1052 | 0.0986 | 0.1122 | 0.0850 | 0.0799 | 0.0899 | 0.0530 | 11964 | 2445 | 1816 | 2029 | 1258 | 1016 |
| Ica | 0.4573 | 0.4428 | 0.4709 | 0.2060 | 0.2013 | 0.2107 | 0.2893 | 0.2810 | 0.2976 | 0.2693 | 0.2601 | 0.2790 | 0.1260 | 0.1179 | 0.1344 | 0.0677 | 18965 | 8672 | 3907 | 5486 | 5107 | 2389 |
| Junin | 0.3249 | 0.3094 | 0.3415 | 0.1821 | 0.1777 | 0.1869 | 0.1993 | 0.1931 | 0.2062 | 0.1714 | 0.1644 | 0.1794 | 0.1222 | 0.1159 | 0.1291 | 0.0506 | 20723 | 6732 | 3774 | 4130 | 3552 | 2531 |
| La Libertad | 0.4715 | 0.4557 | 0.4872 | 0.1681 | 0.1637 | 0.1729 | 0.2717 | 0.2638 | 0.2797 | 0.2873 | 0.2762 | 0.2989 | 0.2003 | 0.1907 | 0.2108 | 0.0467 | 27553 | 12991 | 4632 | 7485 | 7914 | 5518 |
| Lambayeque | 0.4849 | 0.4703 | 0.4992 | 0.1867 | 0.1821 | 0.1915 | 0.2392 | 0.2319 | 0.2469 | 0.3361 | 0.3248 | 0.3466 | 0.1856 | 0.1742 | 0.1973 | 0.0655 | 26448 | 12824 | 4937 | 6326 | 8888 | 4909 |
| Lima | 0.4958 | 0.4827 | 0.5088 | 0.1835 | 0.1795 | 0.1876 | 0.3089 | 0.3016 | 0.3164 | 0.3027 | 0.2937 | 0.3120 | 0.1763 | 0.1686 | 0.1844 | 0.0684 | 234575 | 116298 | 43036 | 72455 | 70996 | 41357 |
| Loreto | 0.1980 | 0.1732 | 0.2226 | 0.1515 | 0.1459 | 0.1571 | 0.2034 | 0.1912 | 0.2150 | 0.0481 | 0.0445 | 0.0516 | 0.0903 | 0.0796 | 0.1012 | 0.0941 | 23061 | 4565 | 3494 | 4690 | 1110 | 2082 |
| Mdr Dios | 0.5052 | 0.4870 | 0.5221 | 0.1742 | 0.1682 | 0.1799 | 0.3010 | 0.2914 | 0.3113 | 0.3828 | 0.3667 | 0.3981 | 0.0872 | 0.0820 | 0.0928 | 0.0980 | 3933 | 1986 | 684 | 1183 | 1505 | 343 |
| Moquegua | 0.5232 | 0.5084 | 0.5372 | 0.1937 | 0.1886 | 0.1983 | 0.3025 | 0.2933 | 0.3118 | 0.2821 | 0.2723 | 0.2919 | 0.2687 | 0.2570 | 0.2800 | 0.0798 | 4817 | 2520 | 932 | 1456 | 1359 | 1294 |
| Pasco | 0.1863 | 0.1717 | 0.2012 | 0.1985 | 0.1939 | 0.2035 | 0.1791 | 0.1729 | 0.1852 | 0.0783 | 0.0744 | 0.0821 | 0.0145 | 0.0137 | 0.0153 | 0.0519 | 4227 | 787 | 839 | 756 | 331 | 61 |
| Piura | 0.4426 | 0.4276 | 0.4568 | 0.1645 | 0.1602 | 0.1687 | 0.2755 | 0.2676 | 0.2833 | 0.2296 | 0.2209 | 0.2384 | 0.2034 | 0.1945 | 0.2128 | 0.0746 | 43865 | 19413 | 7217 | 12083 | 10071 | 8923 |
| Puno | 0.2843 | 0.2692 | 0.3000 | 0.1251 | 0.1216 | 0.1285 | 0.1780 | 0.1724 | 0.1840 | 0.2671 | 0.2581 | 0.2771 | 0.0101 | 0.0096 | 0.0108 | 0.0717 | 28725 | 8166 | 3592 | 5113 | 7672 | 291 |
| San Martin | 0.2231 | 0.2030 | 0.2449 | 0.1789 | 0.1731 | 0.1848 | 0.2312 | 0.2232 | 0.2404 | 0.0000 | 0.0000 | 0.0000 | 0.0949 | 0.0833 | 0.1066 | 0.0619 | 14870 | 3317 | 2659 | 3438 | 0 | 1411 |
| Tacna | 0.5313 | 0.5164 | 0.5454 | 0.1458 | 0.1410 | 0.1509 | 0.4025 | 0.3937 | 0.4112 | 0.2625 | 0.2504 | 0.2756 | 0.2122 | 0.2012 | 0.2235 | 0.0948 | 10781 | 5728 | 1572 | 4339 | 2830 | 2287 |
| Tumbes | 0.4247 | 0.4066 | 0.4398 | 0.1932 | 0.1879 | 0.1982 | 0.1778 | 0.1701 | 0.1848 | 0.2866 | 0.2755 | 0.2976 | 0.1777 | 0.1645 | 0.1896 | 0.0954 | 6684 | 2838 | 1291 | 1188 | 1915 | 1187 |
| Ucayali | 0.3305 | 0.3138 | 0.3481 | 0.1534 | 0.1490 | 0.1579 | 0.2395 | 0.2325 | 0.2473 | 0.2504 | 0.2381 | 0.2628 | 0.0000 | 0.0000 | 0.0000 | 0.0519 | 7328 | 2421 | 1124 | 1754 | 1834 | 0 |

# **Supplementary Figure 1: Population attributable fraction (PAF, %) and absolute number of diabetes cases in 2017 attributable to high body mass index (BMI) in 2014 in women versus men by region in Peru.**

Red colour represents Lima and Callao; blue colour represents the rest of the cities in the coast; brown colour represents cities in the highlands whereas green colour represents cities in the Amazon region.


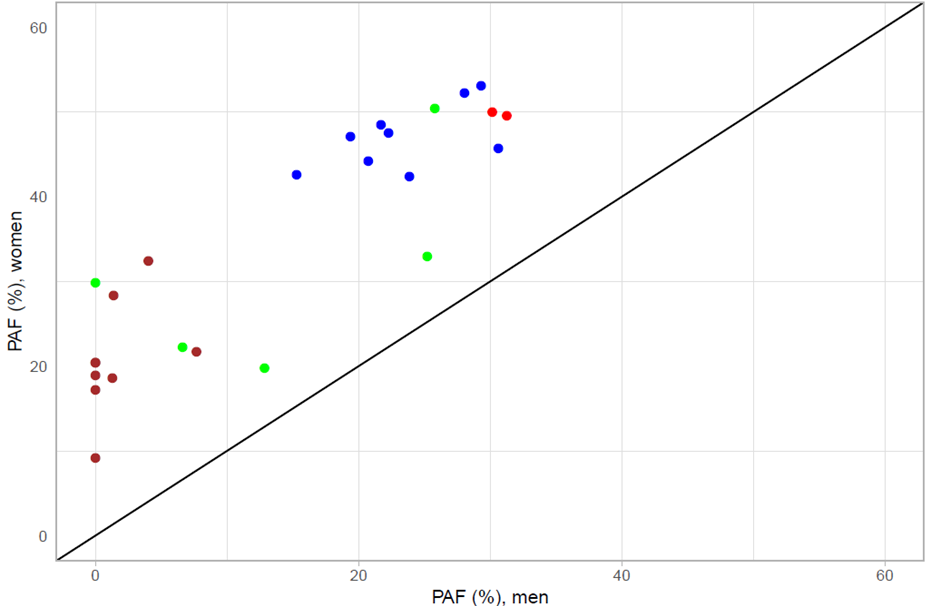


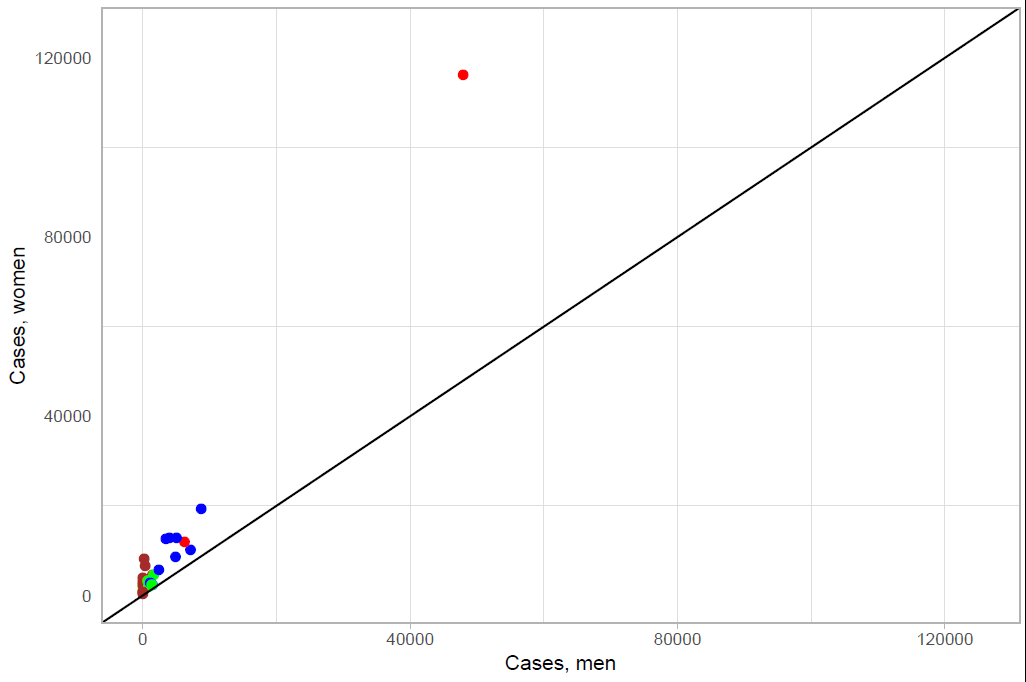

Supplement: Supplementary data 1 [file mmc1.docx]
